# Supplementary material for: Predicting disease risk areas through co-production of spatial models: The example of Kyasanur Forest Disease in India’s forest landscapes
Source: PLoS Negl Trop Dis. 2020 Apr 7;14(4):e0008179. doi: 10.1371/journal.pntd.0008179 (PMC7164675; doi:10.1371/journal.pntd.0008179)
Supplement: S1 Fig — (DOCX) [file pntd.0008179.s001.docx]

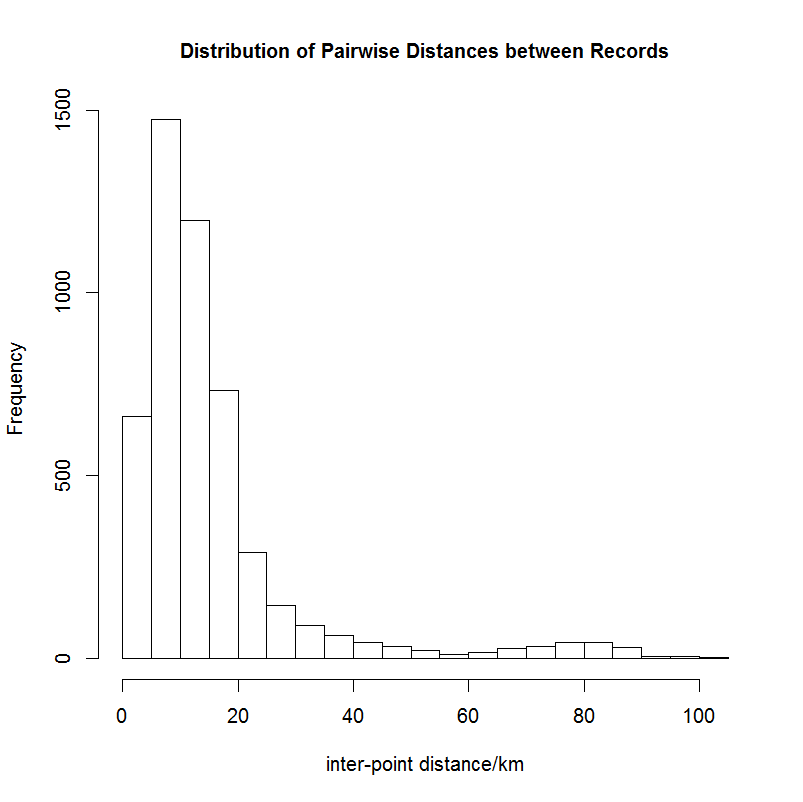


S1 Figure. The distribution of pairwise distances between records of human cases of Kyasanur Forest Disease (2014-2018)
