# Supplementary material for: Predicting disease risk areas through co-production of spatial models: The example of Kyasanur Forest Disease in India’s forest landscapes
Source: PLoS Negl Trop Dis. 2020 Apr 7;14(4):e0008179. doi: 10.1371/journal.pntd.0008179 (PMC7164675; doi:10.1371/journal.pntd.0008179)
Supplement: S1 File — (DOCX) [file pntd.0008179.s002.docx]

**S1 File. Scale of daily movements of people from their households into the forest for livelihood activities in a KFD-affected district**

As part of pilot work for the full MonkeyFeverRisk household survey, in April 2018 the project Research Assistant (M. Rahman) visited six villages in Wayanad District, previously affected by Kyasanur Forest Disease (KFD) to conduct discussions in randomly selected households, with household members about the impacts of KFD on their livelihoods and forest usage and to test the main survey instrument. Respondents including graziers, forest watchers, tea estate and other plantation workers and tribal groups harvesting non-timber forest products, well as forest officers, tribal welfare and health workers. As a parallel activity, to understand scaling of daily movements of people into the forest, respondents were additionally asked how far they moved from their households into the forest for livelihood activities and how this varied with season. The table below presents the results from these discussions. During the KFD risk period from January to March, people forest users move mostly between 1 and 4 km through forest habitats from their homes. In April to May, people travel further into the forest up to 4 or 5 km or more on their daily routes but this is curtailed in June by the rainy season where daily distances travelled drop back down to 1 or 2km.

S4 Table. Reported distances people move from their households into the forest during their daily livelihood activities in different months in a KFD-affected landscape in Wayanad District, Kerala

| Village name |  | Distances between households and forests or plantation (in metres) | Reported distances covered by daily routes from households into the forest each month in km | | | | | |
| --- | --- | --- | --- | --- | --- | --- | --- | --- |
|  | Number of respondents |  | Jan | Feb | Mar | Apr | May | June |
| Poothadi | 13 respondents  . | 500 m to forest and 200 m to government plantation | 4 | 1 | 3 | >5 | >5 | 1 |
| Pulpalli | Discussions held in 13 households across both Pulpalli and Mullenkolly villages that are adjacent | 800 m and 400 m from two different colonies to government plantation | 3 | 2 | >5 | 5 | 4 | 2 |
| Mullenkolly |  | 100 m from Kolavalli colony to forest  2000m from forest to Seethamount | 2 | 3 | 3 | 5 | 4 | 1 |
| Chethalayam | 15 respondents involved in collective grazing | 400 to 700 m | 2 | 3 | 4 | 4 | >5 | 2 |
| Chuiiyode | 2 tea estate workers | 200 to 800 m | 1 | 2 | 4 | 3 | <5 | 1 |
| Appapara | 21 households | 600 m | 2 | 3 | 3 | 5 | 5 | 2 |
