# Supplementary material for: Predicting disease risk areas through co-production of spatial models: The example of Kyasanur Forest Disease in India’s forest landscapes
Source: PLoS Negl Trop Dis. 2020 Apr 7;14(4):e0008179. doi: 10.1371/journal.pntd.0008179 (PMC7164675; doi:10.1371/journal.pntd.0008179)
Supplement: S2 File — (DOCX) [file pntd.0008179.s003.docx]

**S2 File. Participatory methods to identify with cross-sectoral stakeholders the key risk factors for Kyasanur Forest Disease and key policies affecting transmission and management.**

The participatory MonkeyFeverRisk Framing workshop was held on 16th August 2018 in Bengaluru, Karnataka, India. It involved over 20 experts from different KFD-affected districts and state level officials of Karnataka, Maharashtra and Kerala from the public and animal health, agriculture, forestry and social welfare sectors [1]. Participants of the workshop were selected based on a stakeholder mapping exercise carried out by the project team, in which key actors from different sectors and working at different scales were identified as playing major roles in terms of the understanding and management of KFD. The two aims of the workshop were to (i) identify the key risk factors for KFD as prioritized by stakeholders and (ii) identify key policies that affect KFD transmission and management using participatory approaches.

For (i), the first step involved participants independently identifying key risk factors which they deemed important. As a second step, facilitators compiled the information provided by individual participants and arrived at a comprehensive list of risk factors, which were presented independently for ease of ranking. As the third step, the participants ranked the risk factors of their choice (each participant was provided with 5 points/votes to use as they wished in ranking the identified risks, i.e a single risk factor could be given 5 votes or the votes could be distributed over different risk factors). This enabled the ranking and shortlisting of key risk factors (step 4).

For (ii) policy impacts on KFD, stakeholders were divided into 5 groups and asked to address “How do you see national and state level policies affecting KFD management?” while a facilitator recorded and verified key discussion points on a flip chart.

**References**

1. Young J, Prashanth NS, Savatagi SB, Oommen MA, Mujeeb R, Kiran SK, et al. OptimizingForest Benefits whilst Minimizing Impacts of Kyasanur Forest Disease and OtherZoonotic Diseases: Report of the Stakeholder Workshop, 16th August 2018, Bengaluru, India. MonkeyFeverRisk project; 2018 Dec p. 24**.**
