# Supplementary material for: Predicting disease risk areas through co-production of spatial models: The example of Kyasanur Forest Disease in India’s forest landscapes
Source: PLoS Negl Trop Dis. 2020 Apr 7;14(4):e0008179. doi: 10.1371/journal.pntd.0008179 (PMC7164675; doi:10.1371/journal.pntd.0008179)
Supplement: S3 File — (DOCX) [file pntd.0008179.s004.docx]

**S3 File. Environmental predictors of Kyasanur Forest Disease distribution**

This file details the sources and processing of environmental predictors offered to the statistical analysis. All processing was performed in the raster package [1] of the R program [2] unless otherwise specified, with function names as specified below.

**Topography predictors**

Elevation data was extracted in tiles from Shuttle Radar Topography Mission data version 4 [3] an original resolution of 0.000833 degrees Latitude and Longitude resolution (approximately 90m by 90m grid cells). Tiles were mosaicked across the study region using the merge function. A slope value for each pixel was calculated (in degrees) using the terrain function of the raster package, and a focal window of 3 by 3 cells. Both the resulting elevation and slope rasters were cropped to the administrative boundaries of the Shivamogga District (raster package: crop function) and re-projected to an equal area projection (Albers equal area conic projection) using the projectRaster function (method=”bilinear”). Mean elevation and slope values were then calculated across the study 1km and 2km grid cells, using the aggregate function to average values across the appropriate number of ~90m grid cells and then the resample function to align the resulting grid to the study grids (Fig. S2A).

**Landscape predictors**

Metrics of the current availability (and fragmentation) of forest, agricultural and built-up land use types as well as that of water-bodies were extracted from the MonkeyFeverRisk Land Use Land Cover map of Shimoga. The latter was produced from classification of earth observation data from 2016 to 2018 using the methods described in the Supplementary information S3 file. The LULC map had an original grid square resolution of 0.000269 degrees Latitude and Longitude resolution (or 30m x 28m grid cells) and nine different LULC classes (Table S3). It was cropped to the administrative boundaries of the Shimoga District (raster package: crop function) and re-projected to the equal area projection (Albers equal area conic projection) using the projectRaster function (method=”ngb” for categorical data). The agriculture and fallow land classes were combined before landscape analysis (due to the difficulty of separating them accurately in the classification process, Table S3).

An algorithm was developed in R to identify which of the pixels in the LULC map coincided with each 1km and 2km grid cell of the study area. The ClassStat function of the SDM Tools package [4] was used to calculate the proportional area of each 1km or 2km grid cell landscape that was made up of a particular land class, as well patch density and edge density metrics for the forest classes as indicators of fragmentation and forest-agriculture interface habitat respectively (Fig. S2B). The proportional area values (*p_i_*) of the *n* different forest classes (wet evergreen forest, moist deciduous forest, dry deciduous forest and plantation) were used to calculate an index of forest type diversity per grid cell as follows, after Shannon & Weaver (1949) [5]:

$$H^{'}= -\sum_{1}^{n} p_{i}(\log_{n} p_{i})$$

Using the same formula, the proportional area values of all LULC classes were used to calculate an index of land use type diversity per grid cell across the study region.

Metrics of longer term forest changes in Shimoga since 2000 were derived from a global product by Hansen et al. (2013) [6] available at a spatial resolution of 1 arc-second per pixel, (~ 30 meters per pixel at equator). Forest loss during the period 2000–2014, is defined as a stand-replacement disturbance, or a change from a forest to non-forest state, encoded as either 1 (loss) or 0 (no loss). Forest gain during the period 2000–2012, is defined as a non-forest to forest change entirely within the study period, encoded as either 1 (gain) or 0 (no gain).


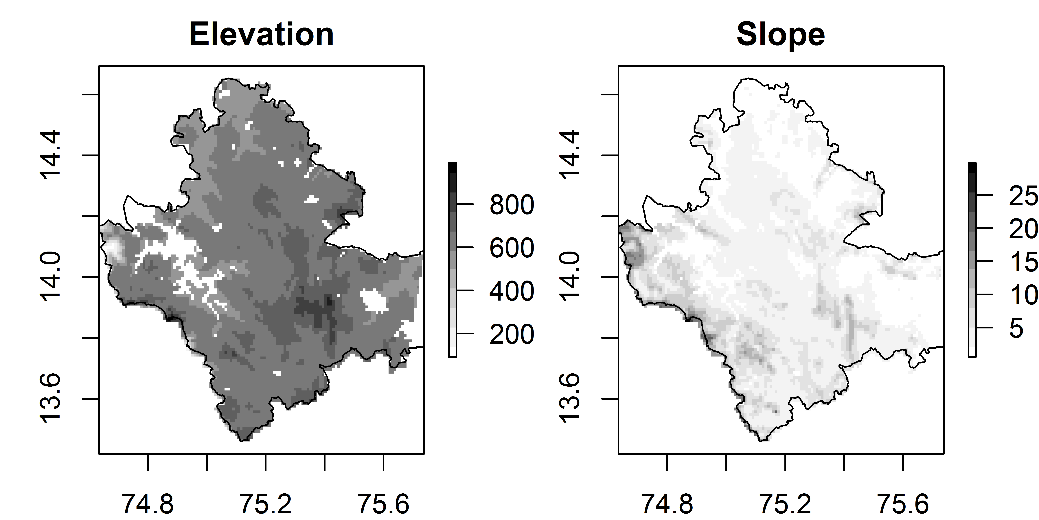


Fig. S2A Mean elevation and slope across Shimoga at a 1km resolution The administrative boundary dataset is from HindudstanTimesLabs (https://github.com/HindustanTimesLabs/shapefiles/), reproduced under the MIT License.


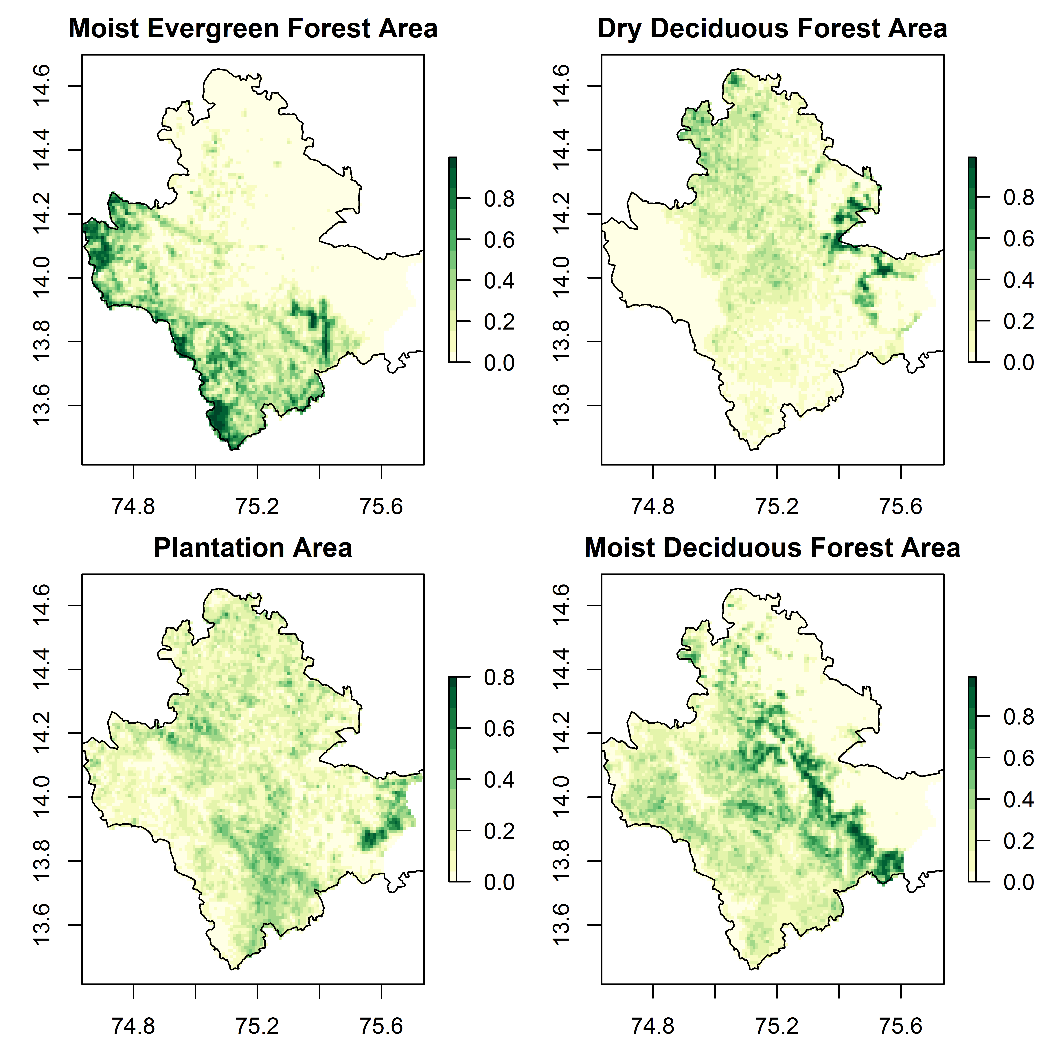


Fig. S2B. Landscape predictors used in BRT models of Kyasanur Forest Disease. The administrative boundary dataset is from HindudstanTimesLabs (https://github.com/HindustanTimesLabs/shapefiles/), reproduced under the MIT License.

These are area of moist evergreen forest, dry deciduous forest, plantation and moist deciduous forest.

These layers were again cropped to the administrative boundaries of the Shimoga District (raster package: crop function) and re-projected to an equal area projection (Albers equal area conic projection) using the projectRaster function (method=”ngb”) in R. An algorithm was developed in R to identify which of the pixels in the loss and gain rasters coincided with each 1km and 2km grid cell of the study area. The ClassStat function of the SDM Tools package [4] was used to calculate the proportional area of each 1km or 2km grid cell that was made up of loss pixels or gain pixels. Forest gain and loss are very highly correlated (r=0.986) and occur in similar places in the landscape (Fig. S2C). Forest loss was a much more common transition than a forest gain affecting 1.2% of land pixels rather than 0.16% of land pixels for forest gain.

To assess how forest loss or gain from a global product like Hansen et al. (2013) should be interpreted locally in south India, we analysed how the loss and gain pixels from Hansen et al. 2013 coincided with classes in the MonkeyFeverRisk LULC map (by extracting the value of the LULC map for the centroids of loss or gain pixels).

The distribution of loss and gain pixels across forest classes from the MonkeyFeverRisk LULC map is shown in Table S2. Locations categorised as a loss by Hansen et al. were most commonly classified currently as plantation, followed by moist evergreen forest, followed by

moist or dry deciduous forest by the MonkeyFeverRisk LULC map. The pattern was similar for the gain pixels. Since not all forest loss pixels were non-forest in the current day and not all forest gain pixels were forest in the current day, the precise meaning of the Hansen et al. (2013) forest loss layer was unclear for south India, though we expect that it is at least indicative of areas where the forest has undergone a larger degree of change since 2000.


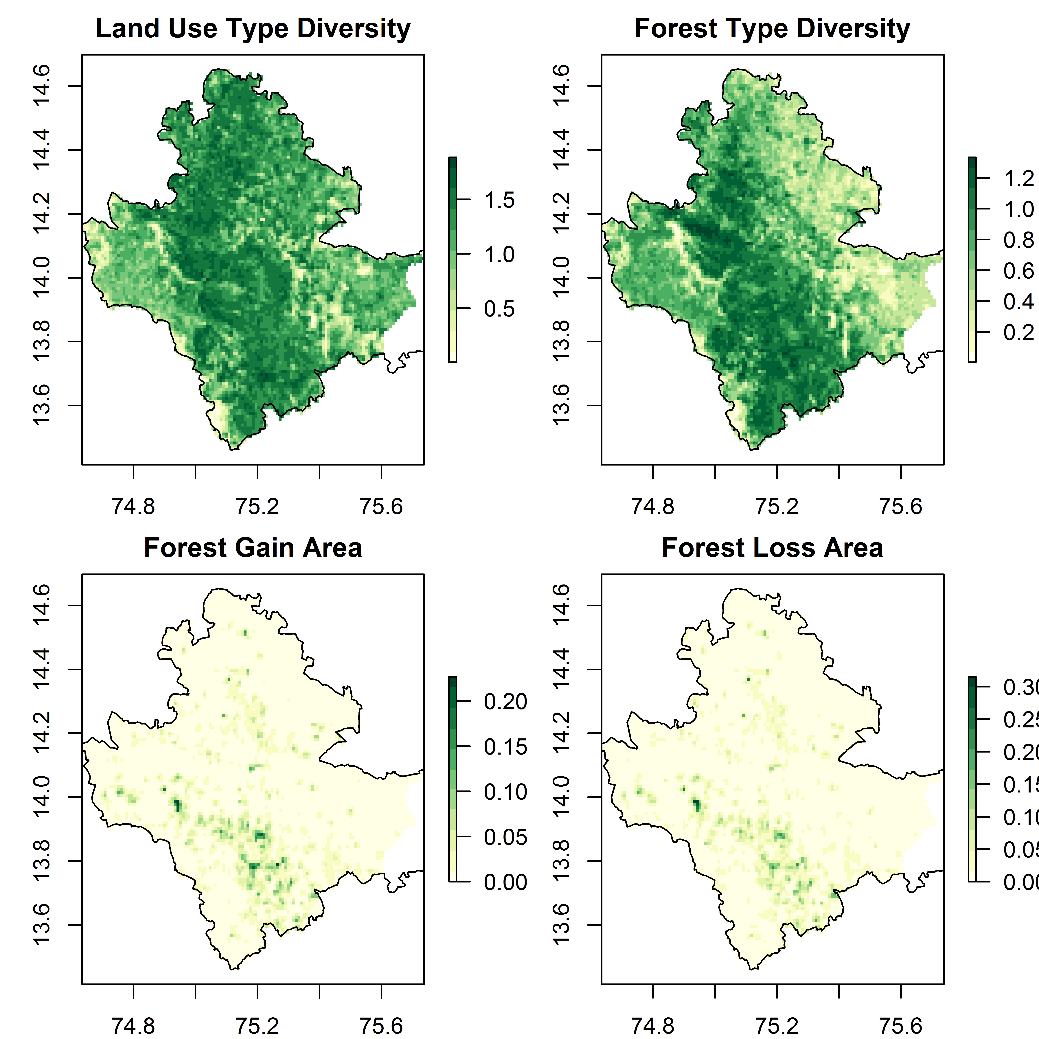


Fig. S2C. Landscape predictors used in BRT models of Kyasanur Forest Disease. These are land use type diversity, forest type diversity, area of forest gain and area of forest loss. The administrative boundary dataset is from HindudstanTimesLabs (https://github.com/HindustanTimesLabs/shapefiles/), reproduced under the MIT License.

Table S2: Percentage of loss (n= 108398) and gain (n= 14646) land pixels from the global Hansen et al. (2013) product that fall into different forest classes according to the MonkeyFeverRisk LULC map

| Land use class | Gain | Loss |
| --- | --- | --- |
| moist evergreen | 30.4 | 26.1 |
| moist deciduous | 6.5 | 16.2 |
| dry deciduous | 3.0 | 9.7 |
| plantation | 46.2 | 37.2 |
| Non-forest classes | 14.0 | 10.9 |

**Host and public health predictors**

Livestock host density data, namely buffalo and indigenous cattle densities in units of total head per village were obtained from Department of Animal Husbandry, Dairying and Fisheries, Government of India Census from 2011 at village level. These were linked to village boundaries from the Survey of India using the village census codes in R. The village areas were calculated from the spatial polygons dataframe of villages using the rgeos package in R, so that the total head per village metrics could be convert into an areal density of buffalo and indigenous cattle per km and then rasterized at 1km and 2km using the rasterize function of the raster package.

The human population size and public health metrics were obtained from the Government of India Population Census 2011. The human population size (census field TOT_P) was again linked to the spatial polygon village boundaries using the census village code (census field VCT_2011) and converted to an areal metric of population density per km and rasterized at 1km and 2km as above. The number of medics per head of population was derived by summing all doctors and para-medicals “in position” across all types of health centres, clinics and dispensaries per village and dividing by the total population of the village (TOT_P) and then linked to village boundaries and rasterized as above. The proximity to health centres was a categorical variable derived from the “Primary.Health.Centre..Numbers” field, where 1 = Primary Health Centre (PHC) within village boundary, 2 = PHC within 5km of village, 3=PHC within 5-10km of village, 4= PHC further than 10km from village. It was linked to village boundaries and rasterized as above.

The resulting raster layers for all predictors were saved in GeoTiff format and then stacked (stack function, raster package), with stacks developed at a 1km grid square resolution and stacks developed at 2km grid square resolution, with and without the forest loss layer.


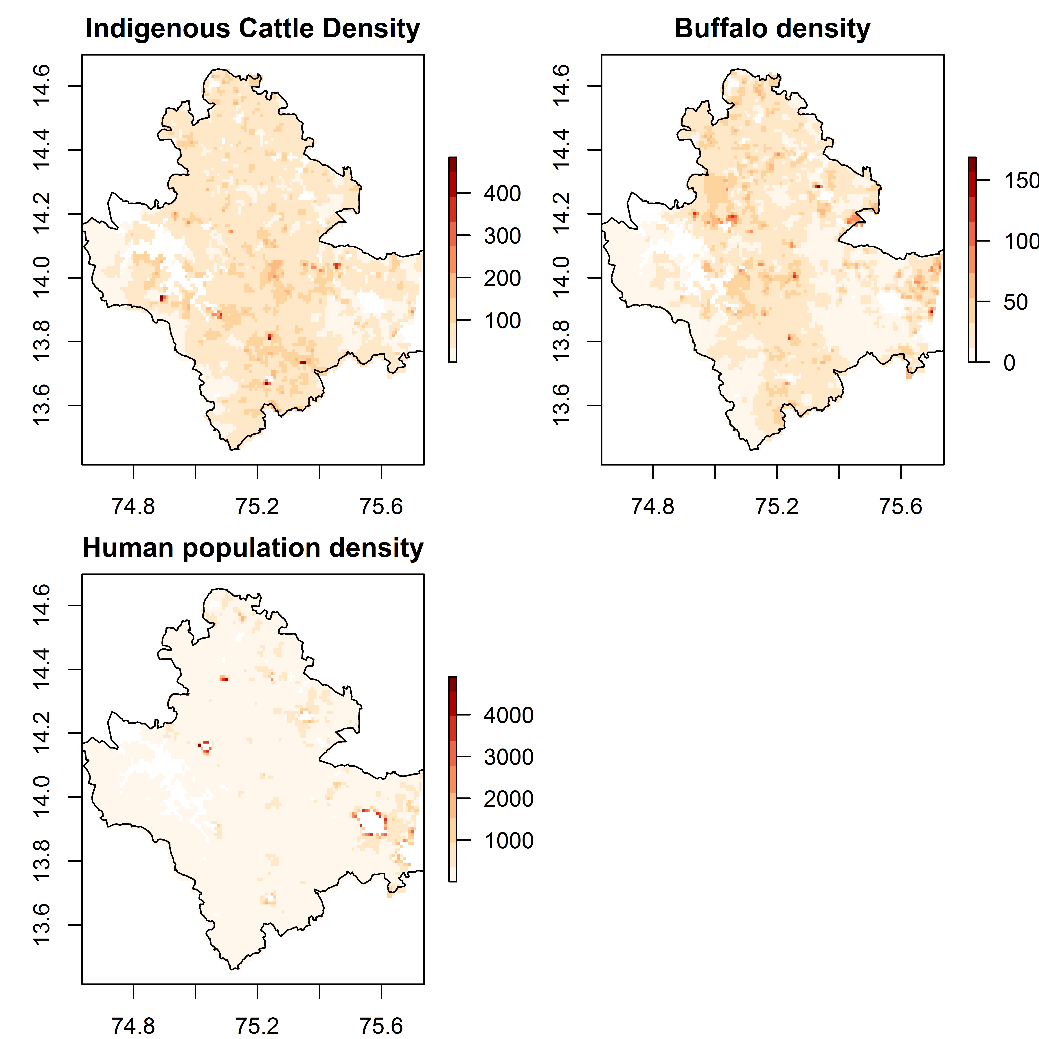


Fig. S2D. Host predictors used in BRT models of Kyasanur Forest Disease.

These are densities (mean head per km) of indigenous cattle, buffalo, and humans. The administrative boundary dataset is from HindudstanTimesLabs (https://github.com/HindustanTimesLabs/shapefiles/), reproduced under the MIT License.


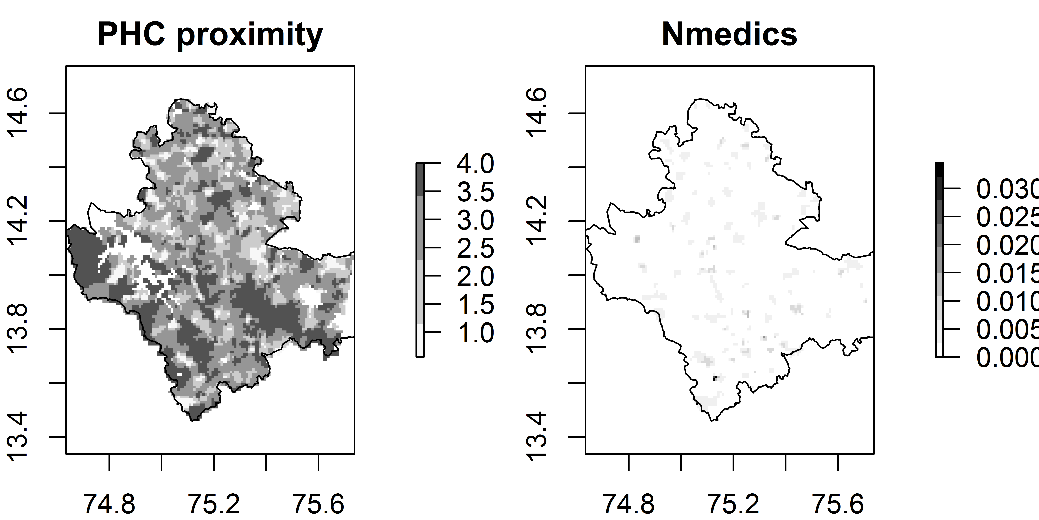


Fig. S1E. Public health predictor used in BRT models of Kyasanur Forest Disease.

These are proximity to a primary health centre (PHC_proximity) and number of medics per head of population per km (Nmedics). The administrative boundary dataset is from HindudstanTimesLabs (https://github.com/HindustanTimesLabs/shapefiles/), reproduced under the MIT License.

**Collinearity**

Prior to the Boosted Regression Tree analysis, collinearity and spatial coherence between pairs of environmental predictors was assessed using Pearson’s correlation analysis and spatial wavelet analysis [8] respectively. The spatial wavelet analysis was carried out on the 500m resolution rasters of the environmental predictors and skill scores were calculated between binary maps of the variables (thresholded using 10% and 25% upper quantiles) at resolutions of 2, 4, 8 pixels i.e. for 1km, 2km, 4km grid cells. Pairs of environmental variables with significant Pearson correlations exceeding r=|0.7|, or with skill scores exceeding 0.75 at one of the pixel resolutions were excluded from the subsequent Boosted Regression Tree analysis.

Land use diversity was excluded because of high Pearson correlation (r=0.788) and coherence with forest diversity, and forest change because of reasonable Pearson correlation (r=0.693) and high coherence with forest loss (and to lesser extent with forest gain). Proximity to Primary Health Centres was retained because although it was highly coherent with forest patch and edge variables, it was not that coherent (< 0.7) with other landscape variables in first three pixel resolutions. The other significant correlation found was between area of plantation and slope (r=0.647) but BRT should be robust to this degree of correlation.

**References**

1. Robert J. Hijmans (2017). raster: Geographic Data Analysis and Modeling. R package version 2.6-7. <https://CRAN.R-project.org/package=raster>
2. R Core Team (2018). R: A language and environment for statistical computing. R Foundation for Statistical Computing, Vienna, Austria.URL <https://www.R-project.org/>
3. Jarvis, A., Reuter, I., Nelson, A., Guevara, E. Hole-filled SRTM for the globe Version 4. 2008.
4. VanDerWal, J., Falconi, L., Januchowski, S., Shoo, L., & Storlie, C. (2014). SDMTools: Species Distribution Modelling Tools: Tools for processing data associated with species distribution modelling exercises. R package version 1.1-221. <https://CRAN.R-project.org/package=SDMTools>
5. Shannon, C. E., and Weaver, W., 1949. The Mathematical Theory of Communication. Urbana: University of Illinois Press.
6. Hansen, M. C., P. V. Potapov, R. Moore, M. Hancher, S. A. Turubanova, A. Tyukavina, D. Thau, S. V. Stehman, S. J. Goetz, T. R. Loveland, A. Kommareddy, A. Egorov, L. Chini, C. O. Justice, and J. R. G. Townshend. 2013. “High-Resolution Global Maps of 21st-Century Forest Cover Change.” Science 342 (15 November): 850–53. Data available on-line from: <http://earthenginepartners.appspot.com/science-2013-global-forest>, accessed November 2017.
7. Bivand, R. & Rundel, C. (2018). rgeos: Interface to Geometry Engine - Open Source ('GEOS'). R package version 0.3-28. <https://CRAN.R-project.org/package=rgeos>
8. Saux Picart, S., Butenschön, M., Shutler, J.D. Wavelet-based spatial comparison technique for analysing and evaluating two-dimensional geophysical model fields. Geosci Model Dev. 2012 Feb 13;5(1):223–30.
