# Supplementary material for: Predicting disease risk areas through co-production of spatial models: The example of Kyasanur Forest Disease in India’s forest landscapes
Source: PLoS Negl Trop Dis. 2020 Apr 7;14(4):e0008179. doi: 10.1371/journal.pntd.0008179 (PMC7164675; doi:10.1371/journal.pntd.0008179)
Supplement: S4 File — (DOCX) [file pntd.0008179.s005.docx]

**S4 File. Deriving the Land Use Land Cover map of Shivamogga from Earth observation data**

The land use land cover (LULC) map of Shivamogga (Fig. S3.1) was derived from Landsat 8 OLI imagery that was composited to produce a single cloud free multi-spectral layer. The OLI imagery used were downloaded from <https://www.usgs.gov/land-resources/nli/landsat/landsat-data-access> as Tier 1 imagery which are atmospherically corrected using [LaSRC](https://landsat.usgs.gov/sites/default/files/documents/lasrc_product_guide.pdf) (<https://landsat.usgs.gov/sites/default/files/documents/lasrc_product_guide.pdf>) and include a cloud, shadow, water and snow mask produced using [CFMASK](https://landsat.usgs.gov/what-cfmask) (<https://landsat.usgs.gov/what-cfmask>, Foga et al 2017), and a per-pixel saturation mask. To cover the whole Shivamogga area, images from the following path‑row combinations were required: 145‑50, 145‑51, 146‑50, and 146‑51. We focussed on using imagery from the dry season (November to February) for two reasons: during this period the spectral difference between deciduous and evergreen forests and other cover types is expected to be greater and cloud cover is less frequent and dense. All individual images with less than 2 % cloud cover that were available for the months November, December, January and February, from January 2016 to February 2018 were selected. In total 10 suitable images were found for path-row 145‑50, 5 for 145‑51, 12 for 146‑50, and 6 for 146‑51. These multiple images were then composited per-pixel to produce a single multispectral image layer for Shivamogga by taking, for each of the 7 bands, the median of the 10, 5, 12 or 6 images respectively. A composited (median) NDVI layer was added as an 8^th^ band to better enable the separation between densely vegetated, sparsely vegetated and non-vegetated surfaces. A widely implemented compositing approach is to apply the maximum NDVI rule (White et al 2014), however this approach favours observations acquired when a pixel contains a higher density of green vegetation. Compositing using the median has been shown to produce imagery that is more representative of the entire time period of the original images (Flood 2013). This composited layer was then classified into 9 classes designed to represent the distinct natural, semi-natural and anthropogenic land use and cover types found within the Shivamogga landscape. Table S3.1 lists the class names with their respective description. We applied a Support Vector Machine classifier (with a Radial Basis Function kernel, a cost value of 1000 and a gamma value of 10-5 ) that was trained and validated using 264 reference polygons collected randomly, mostly along the road network of Shivamogga (Figure S3.2). The reference polygons were split into 181 polygons (i.e ~70%) for training and 83 (i.e. ~30%) for validation. The classification was performed on the Google Earth Engine (GEE) cloud computing platform. Final steps involved (i) a manual editing of the resulting map to include the ‘Shola forest’ class which could not be separated spectrally from the dry deciduous forest, and (ii) the application of a smoothing (3x3 majority) filter to remove single pixel speckle.

The confusion matrix, showing the accuracy of the final LULC map is shown in Table S3.2. The overall accuracy is 0.91. Most classes are classified well with producer’s and consumer’s accuracies ranging between 0.77 to 1.00 and 0.86 to 1.00 respectively. The exceptions are the consumer’s accuracy for ‘Agriculture land’ (i.e. 0.55) and ‘Built-up’ (i.e. 0.67). In both cases this represent a commission error where ‘Fallow land’ is wrongly classified as ‘Agriculture land’ or ‘Built-up’.


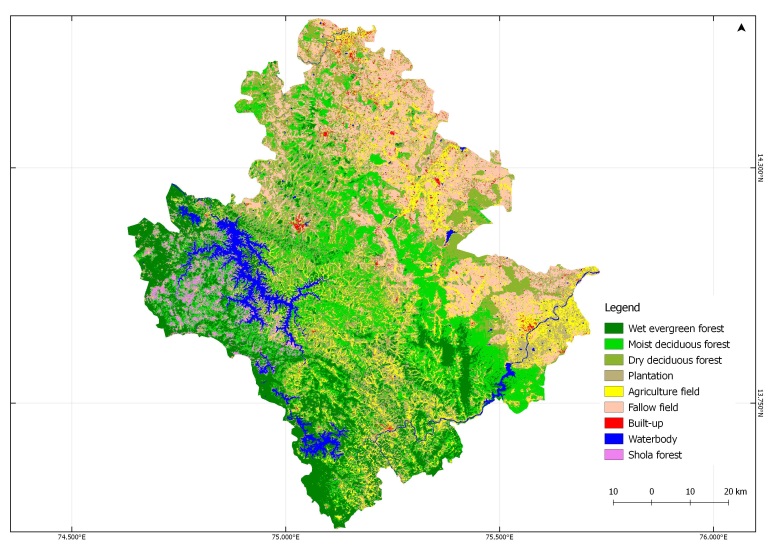


Fig. S3.1: The Shivamogga LULC map. These raster maps are not under copyright since they are a product of this study. The administrative boundary dataset is from HindudstanTimesLabs (https://github.com/HindustanTimesLabs/shapefiles/), reproduced under the MIT License.


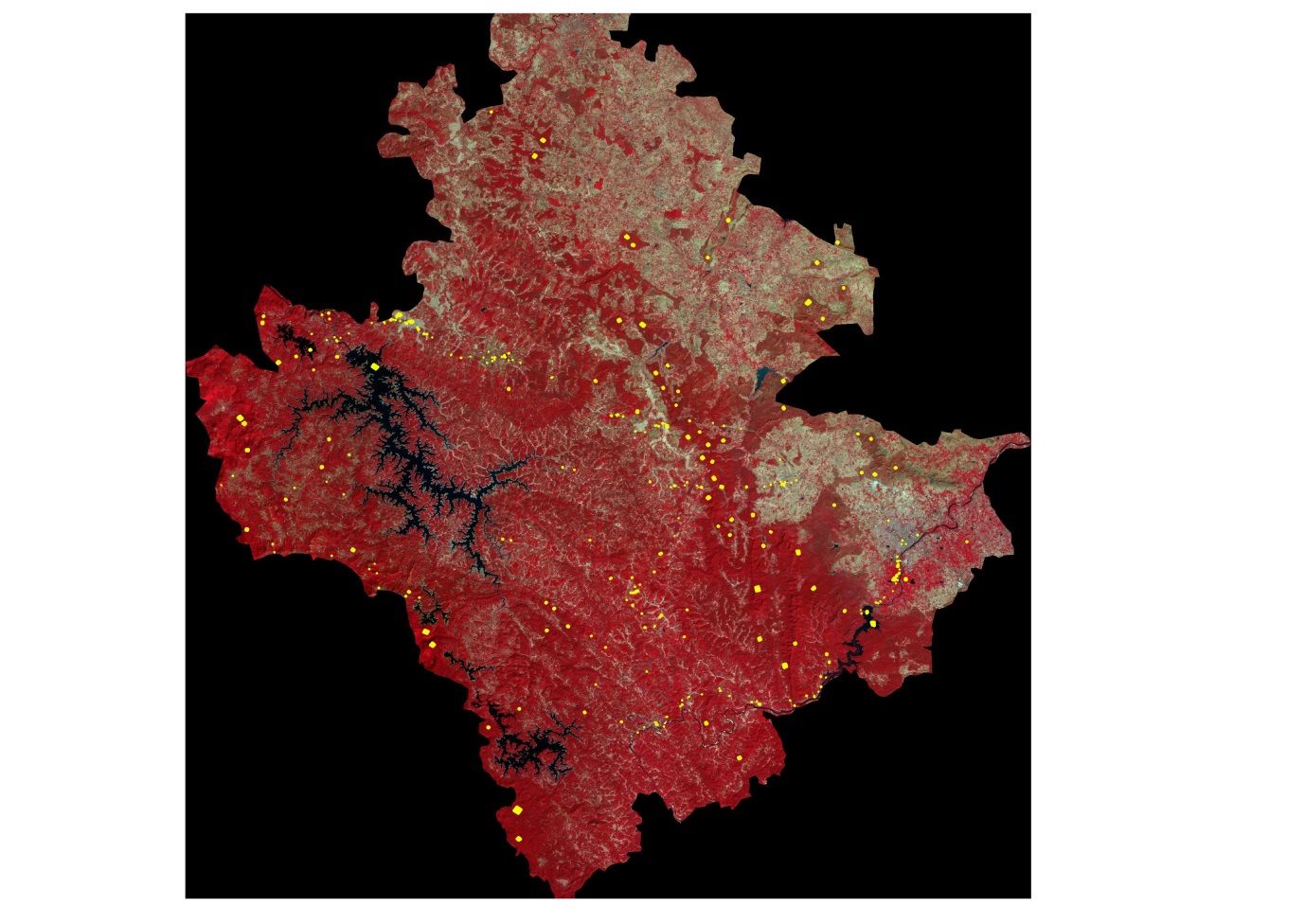


Fig. S3.2: Map of Shivamogga as a false colour composite of Landsat 8 imagery (OLI bands 5,4,3) showing the location of the 264 ground reference polygons (yellow) used to train and validate the Support Vector Machine classifier. These raster maps are not under copyright since they are a product of this study.

Table S3.1: The class names and their description of the Shivamogga LULC map

| Class Id | Class name | Description |
| --- | --- | --- |
| 1 | Wet evergreen forest | Wet evergreen forests are found in the region which receives enormous amounts of rain all year. These trees often rise to a great height before they open out like a cauliflower. The trees in this forest form a tier pattern: shrubs cover the layer closer to the ground, followed by the short structured trees and then the tall variety. They appear to remain green year round. The evergreen forests do not shed leaves.  Major trees species found in evergreen forest are: Dipterocarpus indicus-Diospyros candolleana-Diospyros oocarpatype, Poeciloneuron indicum facies of Dipterocarpus indicus, Diospyros spp.  Wet evergreen forest can wrongly include areas of moist deciduous forest  Dysoxylum malabaricum-Persea macrantha -Kan- forest type, Mesua forrea-Palaquium ellipticum type, Palaquium ellipticum-Poecilloneuron indicum-Hopea panga type |
| 2 | Moist deciduous forest | The trees have broad trunks, are tall and have branching trunks and roots to hold them firmly to the ground. Some of the taller trees shed their leaves in the dry season. There is a layer of shorter trees and evergreen shrubs in the undergrowth.  Moist deciduous is transition forest between Wet evergreen and Dry deciduous forest and so is likely to misclassify as wet evergreen or dry deciduous forest.  Lagerstroemia microcarpa-Tectona grandis-Dillenia pentagyna type. |
| 3 | Dry deciduous forest | Dry deciduous forest region receive less rainfall compare to Wet evergreen and moist deciduous forest. The canopy of the trees does not normally exceed 25 metres. Trees shed their leaves in the dry season.  Dry deciduous can be misclassified as Agriculture or Shola forest due to lower reflectance and can wrongly include areas of moist deciduous forest  Anogeissus latifolia-Tectona grandis-Terminalia tomentosa type. |
| 4 | Plantation | Coffee, Tea,Cashewnut, Coconut, Arecanut, Eucalyptus.Arecanut and Eucalyptus plantation are the most commonly found plantations |
| 5 | Fallow land | Agriculture fallow, mostly Paddy.  Fallow land can wrongly include areas of built-up and stpny, dry waterbodies due to similar spectral signatures or can be wrongly classified as built-up |
| 6 | Agriculture field | Vegetated Paddy(Kharif), Gram(Rabi), Pulses, Jowar, Ragi, Bajra(Kharif), Cotton, Sugarcane, Maize |
| 7 | Waterbody | Dam, Streams, ponds ( Mainly includes permanent water waterbody).  seasonal water bodies are highly likely to have been classed as either fallow land or built-up, as the imagery used is from the dry season. |
| 8 | Built-up | Industrial, Town, Village, Hamlet, road, wasteland  Built-up can wrongly include fallow land, and stony, dry waterbodies or can be misclassified as fallow land |
| 9 | Shola forest | The shola forests are patches of stunted trees found in valleys separated by the grasslands. The shola forests are found in very high hilly areas of Western Ghats.  Shola forests are characterized by frost- and fire-resistant grass species like Chrysopogon zeylanicus, Cymbopogon flexuosus, Arundinella ciliata, Arundinella mesophylla, Arundinella tuberculata, Themeda tremula, and Sehima nervosum.  Shola forest are likely to misclassify as Dry deciduous forest and were therefore included through manual digitising |

Table S3.2: The confusion matrix, overall, user’s and consumer’s accuracies for the Shivamogga LULC map.

References:

USGS (2018) Landsat 8 surface reflectance code (LASRC) product guide, LSDS1368, Version-1, pages 40. https://landsat.usgs.gov/sites/default/files/documents/lasrc_product_guide.pdf

Foga, S., Scaramuzza, P.L., Guo, S., Zhu, Z., Dilley, R.D., Beckmann, T., Schmidt, G.L., Dwyer, J.L., Hughes, M.J., Laue, B. (2017). Cloud detection algorithm comparison and validation for operational Landsat data products. [Remote Sensing of Environment, 194, 379-390](http://doi.org/10.1016/j.rse.2017.03.026). <http://doi.org/10.1016/j.rse.2017.03.026>.

White, J.C.,Wulder, M.A., Hobart, G.W., Luther, J.E.,Hermosilla, T., Griffiths, P.,Coops, N.C., Hall, R. J., Hostert, P.,Dyk, A., Guindon, L. (2014) Pixel-Based Image Compositing for Large-Area Dense Time Series Applications and Science, Canadian Journal of Remote Sensing, 40:3, 192-212, DOI: 10.1080/07038992.2014.945827.

Flood N. (2013) Seasonal Composite Landsat TM/ETM+ Images Using the Medoid (a Multi-Dimensional Median),Remote Sensing, 5(12), 6481-6500; doi:[10.3390/rs5126481](https://dx.doi.org/10.3390/rs5126481).
