# Supplementary material for: Predicting disease risk areas through co-production of spatial models: The example of Kyasanur Forest Disease in India’s forest landscapes
Source: PLoS Negl Trop Dis. 2020 Apr 7;14(4):e0008179. doi: 10.1371/journal.pntd.0008179 (PMC7164675; doi:10.1371/journal.pntd.0008179)
Supplement: S5 File — (DOCX) [file pntd.0008179.s006.docx]

**S5 File. Marginal response plots for key predictors of presence of human cases of Kyasanur Forest Disease.**


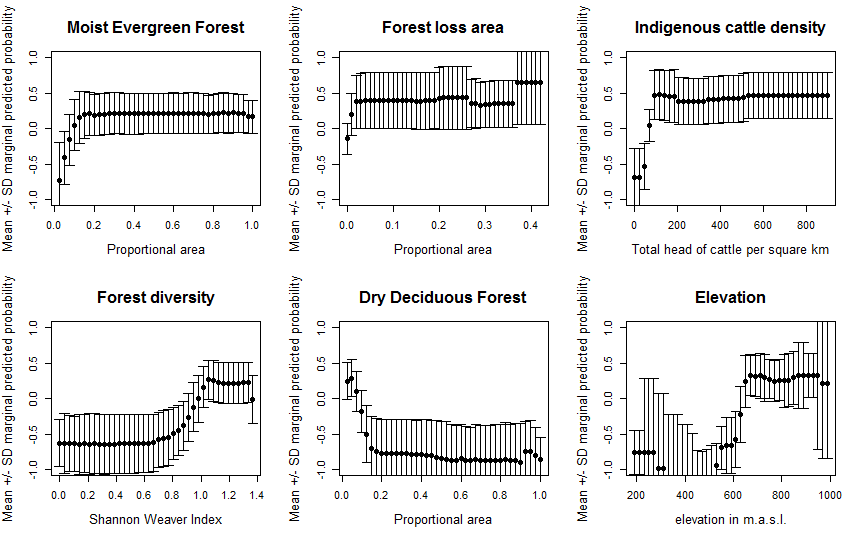


Fig. S5 (a) Marginal response plots for key predictors of presence of human cases of Kyasanur Forest Disease, from models at a 1km resolution (with forest loss as a predictor).
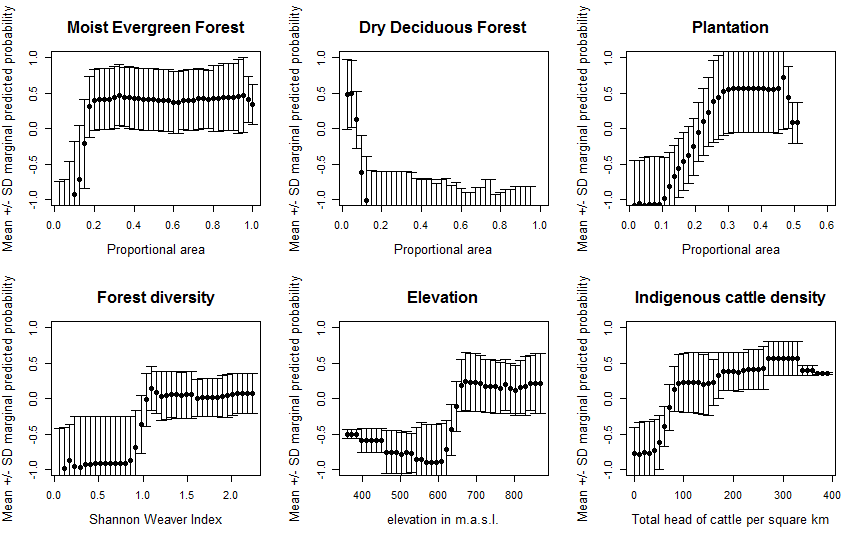
 Fig. S5 (b) Marginal response plots for key predictors of presence of human cases of Kyasanur Forest Disease, from models at a 2km resolution (without forest loss as a predictor).


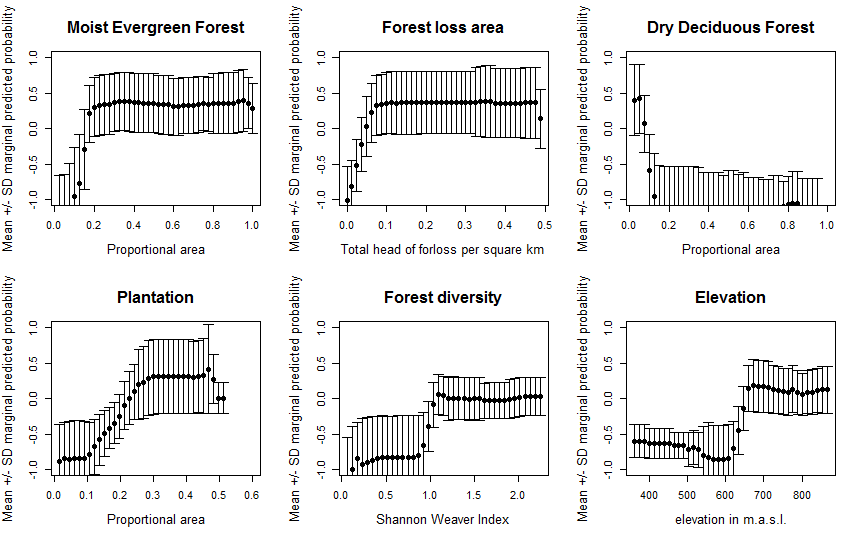
 Fig. S5 (c) Marginal response plots for key predictors of presence of human cases of Kyasanur Forest Disease, from models at a 2km resolution (with forest loss as a predictor).
