# Supplementary material for: Predicting disease risk areas through co-production of spatial models: The example of Kyasanur Forest Disease in India’s forest landscapes
Source: PLoS Negl Trop Dis. 2020 Apr 7;14(4):e0008179. doi: 10.1371/journal.pntd.0008179 (PMC7164675; doi:10.1371/journal.pntd.0008179)
Supplement: S6 File — (DOCX) [file pntd.0008179.s007.docx]

**S6 File. Test for spatial autocorrelation in residuals of Boosted Regression Tree models of Kyasanur Forest Disease**

For models at 1km with and without forest loss among the environmental predictors, significant positive spatial autocorrelation was found in model residuals at 0 to 5km in around 60% of 100 sub-models and at 10 to 15km in only around 30% of 100 sub-models using Moran’s I tests (Table S5 (a) and (b)). This spatial autocorrelation was of low magnitude with average Moran’s I values being around 0.2 in both distance bins.

For models at 2km with and without forest loss among the environmental predictors, significant positive spatial autocorrelation was found in model residuals at 0-5km in around 50% of 100 sub-models and at 10-15km in only around 25% of 100 sub-models using Moran’s I tests (Table S5(c) and (d)). This spatial autocorrelation was of low magnitude with Moran’s I values being around 0.1 between 0 and 5km and around 0.2 between 10 and 15km. Spatial autocorrelation in residuals in distance bins beyond 15km was negative in all model sets.

Given that spatial autocorrelation is of low magnitude and not found consistently across most model runs, we expected this to have a negligible impact on inferred role of environmental predictors and on model accuracy. Furthermore, the model to perform well when validated with independent data (Supplementary Information S8), including that from a different sub-district, which is not to be expected if the quantified disease-environment relationships were dominated by spatial autocorrelation.

Table S6. Moran’s I tests on residuals from BRT models by distance, indicating the average p-values, average significant Moran’s I values, and average number of pairs of sites for each distance bin across model runs, as well as the proportion of runs with significant Moran’s I in each bin. **Bold type** indicates the distance classes where Moran’s I was significant and positive in a substantial proportion of the 100 model runs.

1. Models without forest loss at a 1km resolution

| Distance class |  | |  | | Proportion runs with significant  Moran’s I | | | Number of pairs of sites in distance bin | | | P-value across runs | | | Significant Moran's I values | |
| --- | --- | --- | --- | --- | --- | --- | --- | --- | --- | --- | --- | --- | --- | --- | --- |
|  | Midpoint  km | Upper limit  km | | mean | | s.d. | mean | | s.d. | mean | | s.d. | mean | | s.d. |
| **1** | **2.5** | **5** | | **0.62** | | **0.33** | **112.28** | | **15.52** | **0.08** | | **0.08** | **0.22** | | **0.06** |
| 2 | 7.5 | 10 | | 0.00 | | 0.00 | NA | | NA | NA | | NA | NA | | NA |
| **3** | **12.5** | **15** | | **0.29** | | **0.46** | **82.82** | | **10.30** | **0.19** | | **0.16** | **0.20** | | **0.14** |
| 4 | 17.5 | 20 | | 0.00 | | 0.00 | NA | | NA | NA | | NA | NA | | NA |
| 5 | 22.5 | 25 | | 0.14 | | 0.35 | 51.44 | | 9.09 | 0.22 | | 0.15 | -0.09 | | 0.37 |
| 6 | 27.5 | 30 | | 0.00 | | 0.00 | NA | | NA | NA | | NA | NA | | NA |
| 7 | 32.5 | 35 | | 0.45 | | 0.50 | 40.01 | | 7.50 | 0.13 | | 0.14 | -0.30 | | 0.28 |
| 8 | 37.5 | 40 | | 0.00 | | 0.00 | 60.00 | | NA | 0.19 | | NA | NA | | NA |
| 9 | 42.5 | 45 | | 0.41 | | 0.49 | 33.41 | | 7.68 | 0.14 | | 0.15 | -0.50 | | 0.18 |
| 10 | 47.5 | 50 | | 0.00 | | 0.00 | NA | | NA | NA | | NA | NA | | NA |
| 11 | 52.5 | 55 | | 0.45 | | 0.50 | 21.65 | | 7.59 | 0.11 | | 0.12 | -0.54 | | 0.23 |
| 12 | 57.5 | 60 | | 0.00 | | 0.00 | NA | | NA | NA | | NA | NA | | NA |
| 13 | 62.5 | 65 | | 0.15 | | 0.36 | 9.59 | | 5.27 | 0.20 | | 0.15 | -0.73 | | 0.19 |
| 14 | 67.5 | 70 | | 0.02 | | 0.14 | 3.80 | | 2.59 | 0.11 | | 0.08 | -1.29 | | 0.22 |
| 15 | 72.5 | 75 | | 0.07 | | 0.26 | 4.19 | | 2.84 | 0.28 | | 0.15 | -0.72 | | 0.67 |
| 16 | 77.5 | 80 | | 0.00 | | 0.00 | 1.73 | | 1.01 | 0.38 | | 0.08 | NA | | NA |

Table S6 continued. Moran’s I tests on residuals from BRT models by distance, indicating the average p-values, average significant Moran’s I values, and average number of pairs of sites for each distance bin across model runs, as well as the proportion of runs with significant Moran’s I in each bin. **Bold type** indicates the distance classes where Moran’s I was significant and positive in a substantial proportion of the 100 model runs.

1. Models with forest loss at a 1km resolution.

| Distance class |  | |  | | Proportion runs with significant  Moran’s I | | | Number of pairs of sites in distance bin | | | P-value across runs | | | Significant Moran's I values | |
| --- | --- | --- | --- | --- | --- | --- | --- | --- | --- | --- | --- | --- | --- | --- | --- |
|  | Midpoint  km | Upper limit  km | | mean | | s.d. | mean | | s.d. | mean | | s.d. | mean | | s.d. |
| **1** | **2.5** | **5** | | **0.61** | | **0.33** | 112.28 | | 15.52 | 0.08 | | 0.08 | **0.23** | | **0.06** |
| 2 | 7.5 | 10 | | 0.00 | | 0.00 | NA | | NA | NA | | NA | NA | | NA |
| **3** | **12.5** | **15** | | **0.30** | | **0.46** | 82.83 | | 10.30 | 0.19 | | 0.16 | **0.20** | | **0.14** |
| 4 | 17.5 | 20 | | 0.00 | | 0.00 | NA | | NA | NA | | NA | NA | | NA |
| 5 | 22.5 | 25 | | 0.15 | | 0.36 | 51.44 | | 9.09 | 0.22 | | 0.15 | -0.10 | | 0.35 |
| 6 | 27.5 | 30 | | 0.00 | | 0.00 | NA | | NA | NA | | NA | NA | | NA |
| 7 | 32.5 | 35 | | 0.46 | | 0.50 | 40.01 | | 7.50 | 0.13 | | 0.15 | -0.30 | | 0.28 |
| 8 | 37.5 | 40 | | 0.00 | | 0.00 | 60.00 | | NA | 0.17 | | NA | NA | | NA |
| 9 | 42.5 | 45 | | 0.42 | | 0.50 | 33.41 | | 7.68 | 0.14 | | 0.16 | -0.50 | | 0.17 |
| 10 | 47.5 | 50 | | 0.00 | | 0.00 | NA | | NA | NA | | NA | NA | | NA |
| 11 | 52.5 | 55 | | 0.46 | | 0.50 | 21.65 | | 7.59 | 0.11 | | 0.12 | -0.54 | | 0.23 |
| 12 | 57.5 | 60 | | 0.00 | | 0.00 | NA | | NA | NA | | NA | NA | | NA |
| 13 | 62.5 | 65 | | 0.15 | | 0.36 | 9.59 | | 5.27 | 0.20 | | 0.15 | -0.53 | | 0.77 |
| 14 | 67.5 | 70 | | 0.01 | | 0.10 | 3.80 | | 2.59 | 0.10 | | 0.07 | -1.13 | | NA |
| 15 | 72.5 | 75 | | 0.06 | | 0.24 | 4.19 | | 2.84 | 0.28 | | 0.15 | -0.72 | | 0.73 |
| 16 | 77.5 | 80 | | 0.00 | | 0.00 | 1.73 | | 1.01 | 0.38 | | 0.08 | NA | | NA |

Table S6 continued. Moran’s I tests on residuals from BRT models by distance, indicating the average p-values, average significant Moran’s I values, and average number of pairs of sites for each distance bin across model runs, as well as the proportion of runs with significant Moran’s I in each bin. **Bold type** indicates the distance classes where Moran’s I was significant and positive in a substantial proportion of the 100 model runs.

1. Models without forest loss at a 2km resolution

| Distance class |  | |  | | Proportion runs with significant  Moran’s I | | | Number of pairs of sites in distance bin | | | P-value across runs | | | Significant Moran's I values | |
| --- | --- | --- | --- | --- | --- | --- | --- | --- | --- | --- | --- | --- | --- | --- | --- |
|  | Midpoint  km | Upper limit  km | | mean | | s.d. | mean | | s.d. | mean | | s.d. | mean | | s.d. |
| **1** | **2.5** | **5** | | **0.49** | | **0.37** | **98.67** | | **12.40** | **0.10** | | **0.09** | **0.25** | | **0.08** |
| 2 | 7.5 | 10 | | 0.01 | | 0.10 | 61.00 | | NA | 0.01 | | NA | 0.26 | | NA |
| **3** | **12.5** | **15** | | **0.23** | | **0.42** | **85.64** | | **12.78** | **0.20** | | **0.16** | **0.22** | | **0.07** |
| 4 | 17.5 | 20 | | 0.00 | | 0.00 | NA | | NA | NA | | NA | NA | | NA |
| 5 | 22.5 | 25 | | 0.16 | | 0.37 | 54.74 | | 10.67 | 0.23 | | 0.16 | -0.04 | | 0.32 |
| 6 | 27.5 | 30 | | 0.00 | | 0.00 | NA | | NA | NA | | NA | NA | | NA |
| 7 | 32.5 | 35 | | 0.45 | | 0.50 | 40.57 | | 8.18 | 0.12 | | 0.13 | -0.41 | | 0.20 |
| 8 | 37.5 | 40 | | 0.00 | | 0.00 | NA | | NA | NA | | NA | NA | | NA |
| 9 | 42.5 | 45 | | 0.47 | | 0.50 | 33.03 | | 9.95 | 0.13 | | 0.13 | -0.40 | | 0.18 |
| 10 | 47.5 | 50 | | 0.00 | | 0.00 | NA | | NA | NA | | NA | NA | | NA |
| 11 | 52.5 | 55 | | 0.26 | | 0.44 | 20.13 | | 8.36 | 0.19 | | 0.16 | -0.47 | | 0.25 |
| 12 | 57.5 | 60 | | 0.00 | | 0.00 | 24.00 | | NA | 0.44 | | NA | NA | | NA |
| 13 | 62.5 | 65 | | 0.07 | | 0.26 | 6.88 | | 3.59 | 0.26 | | 0.13 | -0.84 | | 0.24 |
| 14 | 67.5 | 70 | | 0.00 | | 0.00 | NA | | NA | NA | | NA | NA | | NA |
| 15 | 72.5 | 75 | | 0.01 | | 0.10 | 2.66 | | 1.79 | 0.34 | | 0.13 | -1.18 | | NA |
| 16 | 77.5 | 80 | | 0.00 | | 0.00 | 6.00 | | NA | 0.42 | | NA | NA | | NA |

Table S6 continued. Moran’s I tests on residuals from BRT models by distance, indicating the average p-values, average significant Moran’s I values, and average number of pairs of sites for each distance bin across model runs, as well as the proportion of runs with significant Moran’s I in each bin. **Bold type** indicates the distance classes where Moran’s I was significant and positive in a substantial proportion of the 100 model runs.

1. Models with forest loss at a 2km resolution.

| Distance class |  | |  | | Proportion runs with significant  Moran’s I | | | Number of pairs of sites in distance bin | | | P-value across runs | | | Significant Moran's I values | |
| --- | --- | --- | --- | --- | --- | --- | --- | --- | --- | --- | --- | --- | --- | --- | --- |
|  | Midpoint  km | Upper limit  km | | mean | | s.d. | mean | | s.d. | mean | | s.d. | mean | | s.d. |
| **1** | **2.5** | **5** | | **0.50** | | **0.36** | **98.67** | | **12.40** | **0.10** | | **0.09** | **0.24** | | **0.08** |
| 2 | 7.5 | 10 | | 0.01 | | 0.10 | 61.00 | | NA | 0.01 | | NA | 0.26 | | NA |
| **3** | **12.5** | **15** | | **0.24** | | **0.43** | **85.64** | | **12.78** | **0.20** | | **0.15** | **0.22** | | **0.07** |
| 4 | 17.5 | 20 | | 0.00 | | 0.00 | NA | | NA | NA | | NA | NA | | NA |
| 5 | 22.5 | 25 | | 0.16 | | 0.37 | 54.74 | | 10.67 | 0.23 | | 0.15 | -0.01 | | 0.32 |
| 6 | 27.5 | 30 | | 0.00 | | 0.00 | NA | | NA | NA | | NA | NA | | NA |
| 7 | 32.5 | 35 | | 0.46 | | 0.50 | 40.58 | | 8.18 | 0.12 | | 0.13 | -0.39 | | 0.22 |
| 8 | 37.5 | 40 | | 0.00 | | 0.00 | NA | | NA | NA | | NA | NA | | NA |
| 9 | 42.5 | 45 | | 0.47 | | 0.50 | 33.03 | | 9.95 | 0.13 | | 0.13 | -0.40 | | 0.18 |
| 10 | 47.5 | 50 | | 0.00 | | 0.00 | NA | | NA | NA | | NA | NA | | NA |
| 11 | 52.5 | 55 | | 0.25 | | 0.44 | 20.14 | | 8.35 | 0.19 | | 0.16 | -0.47 | | 0.26 |
| 12 | 57.5 | 60 | | 0.00 | | 0.00 | 24.00 | | NA | 0.47 | | NA | NA | | NA |
| 13 | 62.5 | 65 | | 0.07 | | 0.26 | 6.88 | | 3.59 | 0.26 | | 0.13 | -0.86 | | 0.21 |
| 14 | 67.5 | 70 | | 0.00 | | 0.00 | NA | | NA | NA | | NA | NA | | NA |
| 15 | 72.5 | 75 | | 0.01 | | 0.10 | 2.66 | | 1.79 | 0.34 | | 0.13 | -1.18 | | NA |
| 16 | 77.5 | 80 | | 0.00 | | 0.00 | 6.00 | | NA | 0.42 | | NA | NA | | NA |
