# Supplementary material for: Predicting disease risk areas through co-production of spatial models: The example of Kyasanur Forest Disease in India’s forest landscapes
Source: PLoS Negl Trop Dis. 2020 Apr 7;14(4):e0008179. doi: 10.1371/journal.pntd.0008179 (PMC7164675; doi:10.1371/journal.pntd.0008179)
Supplement: S7 File — (DOCX) [file pntd.0008179.s008.docx]

**S7 File. Maps of predicted probability of presence of KFD from different sets of BRT models**


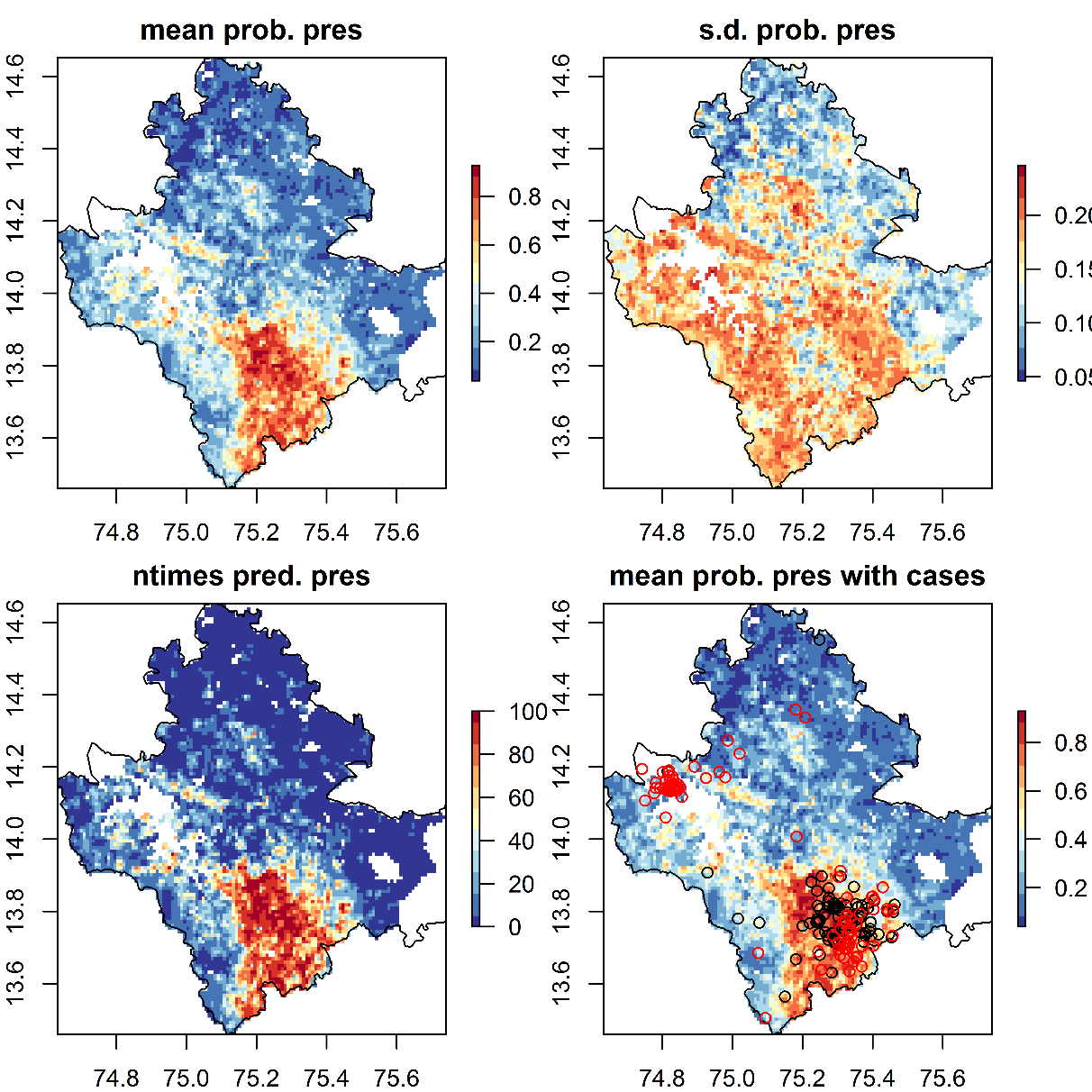


Fig. S7A. Predicted probability of presence of KFD from BRT models containing landscape predictors with forest loss at a 1km resolution. Top panels give mean and standard deviation of the relative predicted probability of presence of KFD in each grid cell across model runs. Top left panel - areas in orange and red have a higher predicted probability of presence of KFD, whilst areas in blue have a lower predicted probability of presence of KFD. Top right panel – areas in yellow and red have more variable predictions of probability of presence of KFD than blue areas. Bottom left panel indicates the number of times KFD is predicted to present in each cell across the 20 model runs, with orange and red indicating that KFD is often predicted to be present. Bottom right panel again depicts the mean predicted probability of presence but the locations of affected households are super-imposed, for the 2014 to 2018 seasons as open black circles and for the 2018 to 2019 season as open red circles. White areas indicate no data. These raster maps are not under copyright since they are a product of this study. The administrative boundary dataset is from HindudstanTimesLabs (https://github.com/HindustanTimesLabs/shapefiles/), reproduced under the MIT License. Human case data are from Department of Health and Family Welfare Services, Government of Karnataka.


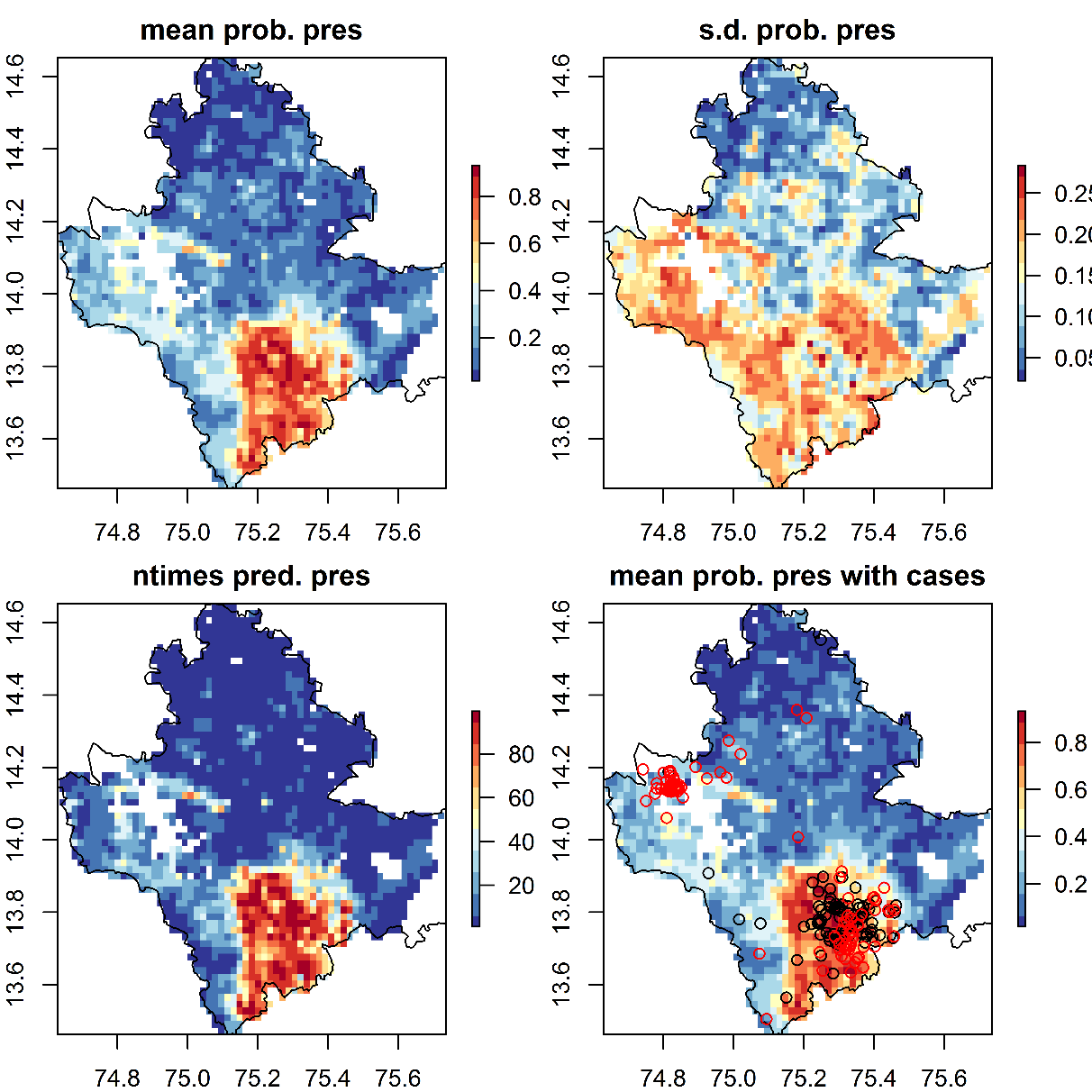


Fig. S7B. Predicted probability of presence of KFD from BRT models containing landscape predictors without forest loss at a 2km resolution. These raster maps are not under copyright since they are a product of this study. The administrative boundary dataset is from HindudstanTimesLabs (https://github.com/HindustanTimesLabs/shapefiles/), reproduced under the MIT License. Human case data are from Department of Health and Family Welfare Services, Government of Karnataka.


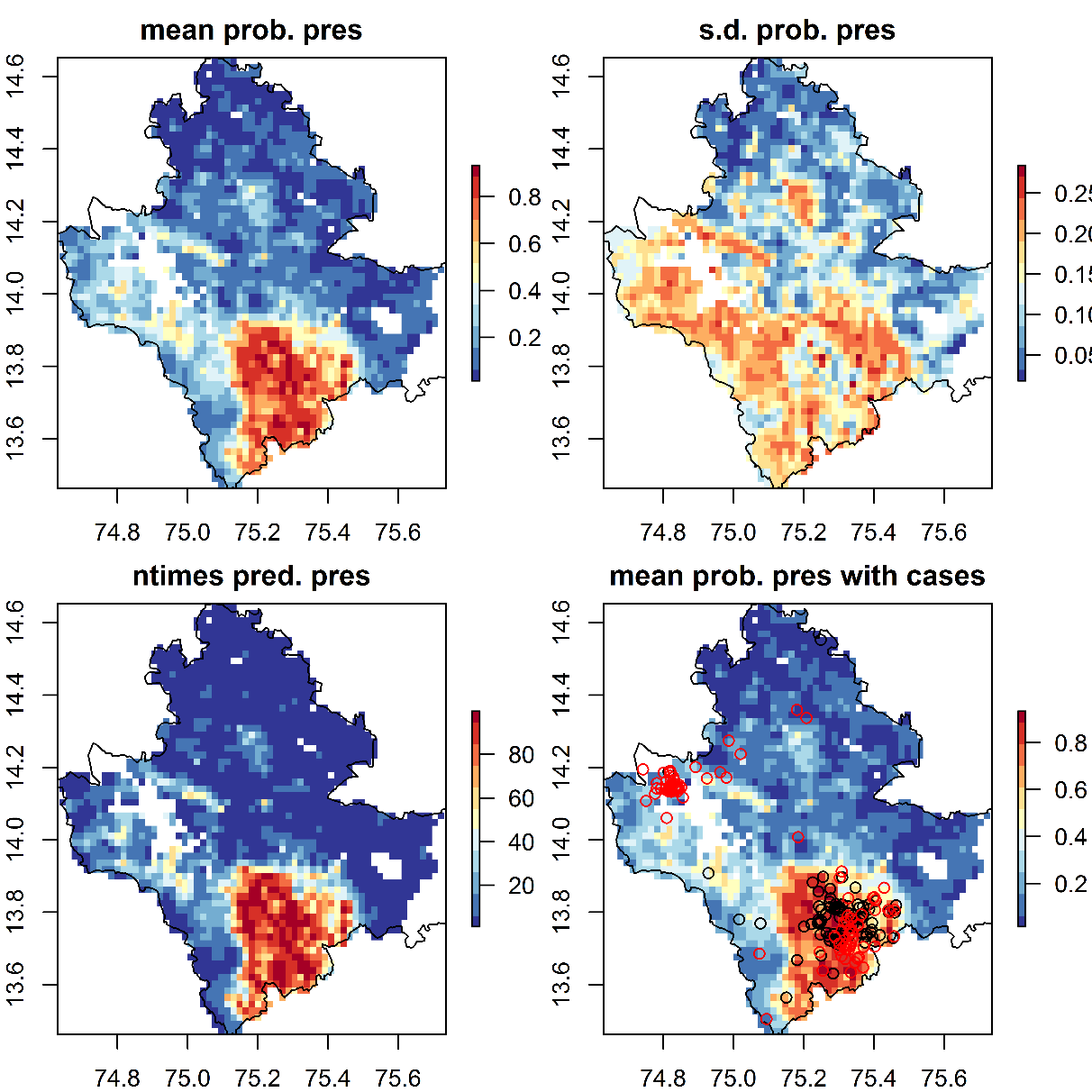


Fig. S7C. Predicted probability of presence of KFD from BRT models containing landscape predictors with forest loss at a 2km resolution. These raster maps are not under copyright since they are a product of this study. The administrative boundary dataset is from HindudstanTimesLabs (https://github.com/HindustanTimesLabs/shapefiles/), reproduced under the MIT License. Human case data are from Department of Health and Family Welfare Services, Government of Karnataka.
