# Supplementary material for: Predicting disease risk areas through co-production of spatial models: The example of Kyasanur Forest Disease in India’s forest landscapes
Source: PLoS Negl Trop Dis. 2020 Apr 7;14(4):e0008179. doi: 10.1371/journal.pntd.0008179 (PMC7164675; doi:10.1371/journal.pntd.0008179)
Supplement: S8 File — (DOCX) [file pntd.0008179.s009.docx]

**S8 File. External validation of the risk maps using 2018/2019 outbreak data and the Boyce Index**


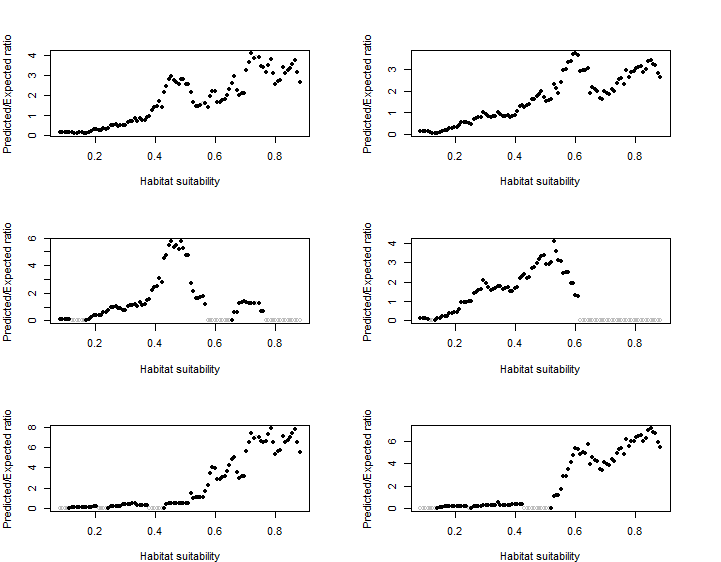


1. Thirthahalli cases
2. Sagara cases
3. All cases

Fig. S8a. Graphs of predicted to expected ratio versus habitat suitability (predicted probability of presence) for models at 1km without forest loss on the left hand side and with forest loss on the right hand side, calculated separately for (a) all cases (b) cases from Sagara taluk only (c) cases from Thirthahalli taluk only.


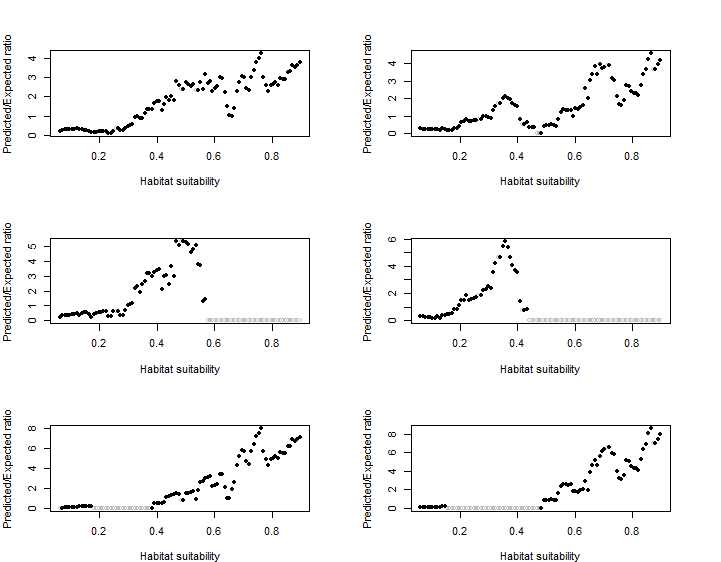


1. Thirthahalli cases
2. Sagara cases
3. All cases

Fig. S8b. Graphs of predicted to expected ratio versus habitat suitability (predicted probability of presence) for models at 2km without forest loss on the left hand side and with forest loss on the right hand side, calculated separately for (a) all cases (b) cases from Sagara taluk only (c) cases from Thirthahalli taluk only.

Table S8a. Boyce Index values for predictive models at 1km for different geographical subsets of the independent test data from the 2018 to 2019 season: The Boyce Index is the Spearman’s correlations between the predicted-to- expected (P/E) ratio and habitat suitability

| Subset of the independent test data  (no. of 1km cells covered by cases) | Models at 1km without forest loss | Models at 1km with forest loss |
| --- | --- | --- |
| All case locations (n = 84 ) | 0.912 | 0.911 |
| Sagara taluk case locations (n= 37) | 0.546 | 0.849 |
| Thirthahalli taluk case locations (n=40) | 0.964 | 0.951 |

Table S8b. Boyce Index values for predictive models at 2km for different geographical subsets of the independent test data from the 2018 to 2019 season: The Boyce Index is the Spearman’s correlations between the predicted-to- expected (P/E) ratio and habitat suitability

| Subset of the independent test data  (no. of 1km cells covered by cases) | Models at 2km without forest loss | Models at 2km with forest loss |
| --- | --- | --- |
| All case locations (n = 68 ) | 0.882 | 0.830 |
| Sagara taluk case locations (n=25 ) | 0.871 | 0.814 |
| Thirthalli taluk case locations (n=36) | 0.938 | 0.925 |
