# Supplementary material for: Predicting disease risk areas through co-production of spatial models: The example of Kyasanur Forest Disease in India’s forest landscapes
Source: PLoS Negl Trop Dis. 2020 Apr 7;14(4):e0008179. doi: 10.1371/journal.pntd.0008179 (PMC7164675; doi:10.1371/journal.pntd.0008179)
Supplement: Attachment — Submitted filename: Reviewers_comments_06012019.docx [file pntd.0008179.s010.docx]

Response to reviewers

We highly appreciate the constructive and thorough comments of the three reviewers. Here we itemise our response to each of the reviewer’s comments in blue under the original comments. The line and page numbers refer to the version with changes highlighted.

Reviewer #1: Objectives are clear and the study design appears appropriate to address the stated objectives. The data used are clearly described and the description of modelling analyses are well written.

Reviewer #2: Authors have analysed the geo-spatial risk factors of an important zoonotic disease using a co-production approach. It has a potential to be of predictive value for focusing public health resources of surveillance for other zoonoses also.

Response: We are very grateful for the positive comments of the reviewers, particularly concerning the applicability of our approach to other zoonoses.

Methods used are mostly appropriate. In the methods section the use of software such as 'R software' and other geo-spatial software should be explicitly mentioned in a separate paragraph.

Response: We feel that it is more helpful for reproducibility of these methods and results to mention the R software and packages at several appropriate points in the section on “Modelling the distribution of Kyasanur Forest Disease with Boosted Regression Trees”, rather than having a separate paragraph with the software details. The details of the R software used for geo-spatial processing are included in the supplementary information file S2. To include these details the main manuscript would cause the manuscript to become very long.

Lines 215-220 and 237-248 (human KFD case data): The exact method used for geo-referencing for the cases of 2018-19 should be mentioned. Whether this involved GIS based geo-coordinate data for each patient or assigning locations based on google-earth/google-map platform after making a filed visit should be specified. The method has been outlined for earlier transmission season. It may be noted that the GIS based method during the field visit (using GIS enabled instruments or even smartphone) is more accurate. The reason for not using a more accurate GIS-based geo-location strategy for the cases (especially for the recent transmission season) should be mentioned. Even for the past cases, GIS based geo-coordinates may be obtained by conducting household visits with the help of the frontline health workers.

Response: We have now clarified in the text (lines 221-227) that the locations of cases were assigned based on Google Earth following personal visits to the households of all affected patients between 2014 and 2017. We then describe how the 2018 and 2019 data geo-location methods differed from the prior years.

Due to the forested and hilly nature of the terrain and remoteness from phone networks, use of smart phones and GPS technology to geo-locate cases is not appreciably more accurate in many locations than marking on Google Earth following a household visit. It is therefore important to have the Google Earth method available for off-line collection of location data.

The impact of accuracy of this key input data on the overall analysis and conclusions should be mentioned in the limitations section (which needs to be added by the authors). This is especially important since the authors themselves discuss the importance of micro-scale level features such as waterbodies in the range of 10 sq metre and the limitations of Landsat image at 30 m scale (lines 304-308)'

Response: The accuracy level of the geo-locations should have limited impact on our analysis since we modelled the distribution of the disease at spatial resolutions of 1km and 2km and selected this resolution to correspond to the scale at which people move for daily activities from their households into the forests (see lines 240-242). Even where environmental factors were measured at micro-scales, we summarised them at the study resolutions of 1km and 2km, before relating them to the distribution of human cases. In fact, as mentioned in the discussion, the vast majority of previous models of patterns in zoonotic diseases have geo-located human cases to the nearest Primary Health Centre. These are often very distant from where exposure occurred and have catchments of 10s of square kilometres. As such, we feel our geo-location at household level and spatial accuracy of at least 300m is a significant step forward in understanding links between environmental conditions and disease. We have added a paragraph acknowledging and explaining this limitation in lines 750-756.

Reviewer #3: This manuscript describes the results of a study of risk factors for Kyasanur Forest Disease in the western Ghats of India. The authors identified potential risk factors to assess in models by involving stakeholders, who also participated in identifying the relevant scales for spatial analyses. The analyses identify several risk factors that are correlated with earlier outbreaks. The authors then identify future outbreak areas and compare these to an outbreak in 2018/19, concluding that the model accurately predicted the location of this outbreak.

R#3: This is an interesting study and the authors are investigating a neglected disease that is a major human health issue. The inclusion of stakeholders is a useful model for conducting an investigation of this type, and the partnership appears to have been a fruitful collaboration among investigators from different areas of research.

Response: We are delighted that the reviewer considers that the co-production approach with stakeholders was a useful model for conducting these types of investigations on zoonotic diseases.

R#3: My major concern with this study is that the authors do not take a critical enough look at their own data, at least based on the general presentation in the manuscript. For example, in several places, they note that the 2018/19 outbreak locations were entirely in zones of high risk, as predicted by their model. This is clearly not the case. Though some of the case locations are within high risk zones, others are in lower risk zones. Further, they indicate, e.g. in the abstract, that they correctly identified a hotspot of an outbreak before it occurred. However, in the region of Sagara taluk depicted in Figure 6, roughly 1/3 of the entire area is identified by the model as high risk, and the outbreak occurred in just one of the many locations they predicted as hotspots. It’s great news that there weren’t more outbreaks, but the authors do need to wrestle with the fact that their risk maps indicated a lot of hotspots, and approximately one of the many regions they identified actually had an outbreak.

Response: We have now extended the validation to the full set of human cases from the 2018 and 2019 season (a 4-fold increase in independent data) and have specifically compared the ability of the models to predict human KFD cases in Tirthahalli, the region from which the past data was drawn, versus in Sagara taluk that had not been affected for over a decade. Accordingly we have taken a more critical look at the predictive ability of the models and moderated our claims on the ability of the model to predict hotspot areas. Specific locations in the manuscript where we have done this include Figs. 1b, Fig 5 and Fig 6 that all depict the larger set of validation data; Supplementary information S8 that includes the new statistical validation results; and the section of the results entitled “Validating predictive models in an outbreak situation” as well as in paragraphs 3 and 4 of the discussion.

How should a resource-poor region deal with predictions like these? Would delivering vaccinations over such a large area strain resources? Might it be possible to refine their models with more/better data so that they could provide more accurate outbreak locations? Is this good enough to be useful? Is it good enough to avoid being counter-productive, e.g. by diffusing a public health response that could have been more focused with better data?

Again, the larger concern here is that the authors risk coming across as advocating a particular approach rather than objectively evaluating its strengths and weaknesses. This is particularly problematic because their approach is not an unequivocal success.

Response: We are very grateful for these interesting and important questions about the use of the predictions. In the third and fourth paragraph of the discussion, we have now specified how the current risk maps could be used to inform management without diffusing health resources and accounting for uncertainty in the models. We have suggested how they could be updated annually to reflect the fact that new cases may extend the estimated environmental niche of human cases.

In another vein, the authors don’t address their inability to develop quantifiable indices for some of the variables identified as important by their stakeholders, e.g. Table 1, nor the fairly coarse fit between their spatial proxies for some of the variables and the identified risk factors. This is a major issue, and should be addressed in the Methods and in the Discussion, particularly in light of their discussion of the advantages of co-production. One of the challenges is that (appropriate) data might not be available. What issues does this raise? What kinds of partnerships could arise from such a discussion, i.e. to collect or gather some of the missing data? Would it be better to wait to run the models until those data can be obtained so that they more accurately reflect actual risk?

Response: We have added a line to the methods to highlight for which risk factors there are not quantifiable indices and then have added the following paragraph to the discussion in lines 778-785 as follows:

*Several risk factors highlighted by stakeholders, including poor data management, low vaccine uptake and under-reporting of monkey deaths by the Forest Department, could not be integrated into models either because they are not measured or because they are poorly linked to available geographical proxies. Awareness of these risk factors still informs treatment of input data, interpretation of model outputs and priorities and partnerships for future collection of both health and environmental data. For example, spatial information on vaccine coverage is now being collected by Primary Health Centres and shared with District Health Officials whilst the Indian Government National Biodiversity Mission aims to address some gaps in data on alternative hosts and vectors for zoonotic diseases.*

We don’t believe that it is better to wait until all those data can be obtained to develop spatial risk models so that they more accurately reflect risk because:

(i) The risk factors mentioned without quantifiable indices are not all inherently spatial (since the stakeholders were asked about overall KFD risk), so will not necessarily affect our model results
(ii) Due to the importance of forest change and structure in the literature and stakeholder knowledge on KFD risk factors, we made significant effort to generate novel fine scale data on land use, forest structure and change to integrate into the analysis. We gained important insights about the linkages between KFD and mosaic forest-paddy-village habitat that would not have been obtained if we had waited for better data.
(iii) Our model predictions show reasonable predictive accuracy for independent validation data, suggesting that the risk factors without quantifiable indices are not making a major contribution at this spatio-temporal scale of analysis
(iv) Given the annual impacts and changes in the disease burdens, there is stakeholder demand for spatial risk maps to improve targeting of surveillance and we have selected an appropriate correlative methods to produce and test these risk maps given the current data availability.

The coarse fit between spatial proxies for some of variables and the identified risk factors, is inevitable when using household level data, and scaling models to the daily movements through the landscape. The difficulties of pinpointing more precisely the locations of exposure and establishing disease-environment relationships at finer spatial scales are discussed in response to reviewer #2 above.

I appreciate the clear explanation of both the need for and the response to spatial clustering of the case presence data, and the supplementary file that provides some additional information. Their conclusion is that there is still mild spatial autocorrelation, based on Moran’s I, but they conclude that it is not important despite its presence in fairly large percentages of their model runs. By what objective grounds do they determine that spatial autocorrelation is not a problem for their analysis? Also, it would be helpful for them to include the sample sizes of case sites after thinning.

Response: The sample size of case sites after thinning is given in line 367 and 368 and is now rephrased to make this clearer to the reader.

The Moran’s I levels were not felt to be a problem because they are low in magnitude and because BRTs are known to be relatively robust to spatial autocorrelation. We had also taken significant steps to remove predictors that were highly correlated in space through our Spatial Wavelet analysis (Supplementary Information S2). Furthermore, if the quantified disease-environment relationships were dominated by spatial components, then one would not expect the model to perform well when validated with independent data, particularly that from a different sub-district.

The authors analyze their data with and without the incorporation of the variable for forest loss. They do not investigate the model with and without any other variables, nor do they rigorously evaluate the potential intercorrelation of their variables. These are important considerations and need to be addressed in the manuscript. In partcular, discussion topics like those on lines 621 and on, in which they interpret what they see as a lack of importance of forest loss as a predictor, are problematic.

Response: Our rigorous analysis of the potential inter-correlation of the variables can be found in Supplementary File S2 of the original submission and combines spatial wavelets and Pearson correlation analysis to remove correlated variables prior to the Boosted Regression Tree Analysis. In the methods, we now direct the reader more clearly to this Supplementary File for information on our treatment of collinearity.

The primary given to forest loss as a predictor, and testing models with and without this factor, was due to the prior knowledge of stakeholders and hypotheses in the literature linking KFD to forest loss. We also wanted to test our model with and without this factor, due to our finding that the global dataset on forest loss did not match with our finer scale local mapping of current day landcover (see Supplementary Information S3). We thought this would be of interest to other epidemiological modellers who may consider using this global data source to link disease patterns to forest loss. Though we do not rigorously evaluate the models with and without other predictors, it should be noted that marginal response plots and % importance already indicate the individual impacts of predictors.

We feel that the discussion of the lack of effect of area of forest loss on the models (Lines 725-739) is sufficiently tentative and highlights the drawbacks in the global datasets which prevent us from making firm conclusions. We have removed the mention of forest loss from the second paragraph of the discussion where the important predictors are listed.

The authors mention in several places (e.g. line 570) in the manuscript that “When the model predictions are overlaid with the outbreak locations in Sagara taluk in Fig. 6 (black locations), it can be seen that all of these locations occur in orange to red areas of high predicted probability of presence (except the south-eastern most isolated location which has a low probability of presence in 2km models).” But this is clearly not the case in any of the maps presented. This undermines this reviewer’s confidence in the objectivity of their analysis.

Response: See response to comments above - the validation and inferences from it have been thoroughly revised.

Reviewer #1: The analysis presented corresponds to the plan presented in the methods and results are clearly presented. In the general comments I make one suggestion with respect to showing the predictive maps without human case locations so that map underneath can be seen - suggest placing in a 4th plot.

Response: We have taken the case locations off the predictive maps as requested and placed them in Fig. 1b and in a 4^th^ plot of Figure 5 as well.

Reviewer #2: Yes

Certain suggestions are as follows

Table 1 - row 8, column 4 - there is overlapping of text with the cell immediately below it. This needs to be corrected. Are the spatial proxies of 'low vaccination coverage' and 'poor diagnosis and surveillance' the same ?

Response: Yes we expect both low vaccination coverage and poor diagnosis and surveillance to be linked geographically to proximity to health centres and availability of health care workers. We have now clarified this in the table text.

Table 2 - the category of predictor can be clubbed and written once instead of repeating it

Response: We have made the requested format change

Table 4 - there is overlap between the last two columns. This needs to be correctly presented.

Response: This has been corrected.

Reviewer #3: The manuscript would be improved by a thorough edit, e.g. line 86.

Response: The manuscript has been edited thoroughly to break up large sentences and improve grammar. For example, line 86 has been edited to remove the additional word noticed by the Reviewer.

Line 374 – Here, the methods indicate that the models are parameterised with presence-only data, though previously absence data were described. Please make a clearer distinction between when presence-only and presence/absence data were used to develop models.

Response: We have rewritten this sentence to clarify that models are developed through out with presence/pseudo-absence data.

Line 275 – “Forest diversity” is not the ideal descriptor for the variable used here. Based on the description, they seem to have used “Diversity of forest types”, while “forest diversity” sounds like they quantified the species diversity within forests, which they didn’t. I would suggest changing this throughout.

Response: We have now altered Table 2 and the results and discussion to clarify that we mean “Diversity of forest types”.

Line 621 – The authors discuss why incorporating forest loss did not substantially improve model accuracy. Their explanations do not incorporate a reason for why forest loss emerged as the second most important factor when it was incorporated. One possibility is that forest loss is correlated with another one of their variables, and I do not see where they rule out this possibility.

Response: Our analysis of collinearity rules out strong univariate correlation between pairs of predictors (in Supplementary Information 2), but the forest loss predictor may be strongly related to particular combinations of the other predictors or to unmeasured variables of importance. Given that models including forest loss performed more poorly in independent validation and the global forest loss layers correspond poorly with current day local land use, we are keen not to over-interpret this finding.

Line 711 – Please spell out acronyms.

Response: We have spelled these out as requested.

Figure 2. Why not replace the cryptic panel headings (e.g. Area_ME) that require a special sentence in the legend with informative headings right in the figure? (e.g. “Area of moist evergreen forest”).

Response: These have been replaced as requested.

Figure 6, line 981 – The authors claim that “The black outbreak locations occur in areas of high probability of presence (orange to red) in all models.” This statement is not actually consistent with the data presented in Figure 6, in which quite a number of the black dots occur in blue or white cells.

Response: See response to R#3 comments above - the validation and inferences from it have been thoroughly revised.

For all figures with scales, more information about the scale values needs to be included.

Response: The units of the predictors are given in Table 2 and are not repeated in the Figures. This can be altered if the Editor feels it is necessary.

Reviewer #1: The discussion is well written, public health relevance is addressed and there is discussion on how the analyses presented can advance understanding of KFD distribution in addition to wider application of the methods used.

Reviewer #2: Conclusions (as mentioned in the discussion section) are supported by the data presented.

However, a brief conclusion section should be added after the discussion.

The limitations of the study have not been described and need to be discussed.

Response: We have added information on the limitations of the study as above. Given that these have lengthened the manuscript overall, we felt it best not to add a conclusion section after the discussion.

Reviewer #3: Overall, this is a potentially useful approach to modeling risk factors for an important disease, and the manuscript has many strengths. The co-production approach clearly had benefits, although it also exposed weaknesses of this approach which should be addressed, as should the weaknesses of their predictive abilities based on model outputs.

Response: We have thoroughly revised our validation and are more circumspect about the predictive abilities of the model outputs. We also acknowledge some of the drawbacks of the co-production approach as pointed out by this reviewer.

Reviewer #2: modifications suggested are as follows:

Longer sentences may be reframed and converted to shorter sentences for better comprehension and readability ( examples - lines 681-684 and 704-709)

Response: We revised the manuscript to remove repetition and to break longer sentences into shorter ones.

All R function names such as 'correlog' may be italicized.

Response: We have not italicized R function names but can do so if this is journal policy.

Space may be added between number and km throughout, i.e. '1km' may be written as '1 km'.

Response: We have not changed this as we feel it makes the resolutions of the analysis less readable.

215 - it is not necessary to write the co-author within the article. Rather, the specific contribution can be elaborated within the section of 'Author contributions'

Response: We have retained this because this person’s role in the health system is relevant to why they were able to visit and geo-locate every affected household.

240 - edit duration as ' 11 December 2018 - 12 January 2019'

Response: We now include the entire season of cases in the validation and have given the new dates in this suggested format.

242, 571 and 575 - kindly add space after 'Sagara'

Response: This has been changed.

596 - 'hosts' should be replaced by 'host-related'

Response: We have changed this to host.

600 - 'inter-play' should be replaced by 'interplay'

Response: This has been changed.

605 - consider replacing sentence fragment as ' Overall, landscapes that were at high risk of emergence of human KFD cases...'

Response: This has been replaced with “Overall, landscapes that were high risk of occurrence of human KFD cases”

610- consider adding i.e. after mosaics,

Response: Not added

631 - consider insertion of 'of' between 'more' and 'an'

Response: Added

657 - consider insertion of 'using' between 'than' and 'aggregated data'

Response: Added “with” to scan better with the rest of the sentence

683 - consider use of more technically valid adjectives such as 'insufficiently predictive' instead of 'poor'

Response: Have dropped the use of “poor”

701 - consider replacing comma by 'of'

Response: Added “of”

704 - use abbreviation 'LMIC' instead (abbreviated already)

Response: Abbreviated as suggested

Reviewer #1: This study aimed to develop spatially-explicit predictive models of Kyansur Forest disease (KFD) occurrence for an endemic region of India. The choice of risk factors for inclusion in models was based on information gained from a workshop with key stakeholders. Machine learning algorithms were used to fit models to human case data. Resulting models were then tested for the ability to identify areas where a new outbreak had occurred, not used for initial model fitting. The modelling identified diverse forest-plantation mosaics with high proportion of moist evergreen forest, high cattle density and elevation above 650m as important risk factors for KFD.

Overall, I think that the manuscript is of publishable quality and suitable for PLoS NTDs. In particular, the combined use of: i) information from key stakeholders to guide modelling and; ii) case data from a new area to validate models is novel with respect to zoonotic diseases relevant to developing countries. I do not have any major concerns with respect to the scientific approach and conclusions made but highlight that my skills and experience are not sufficient to comment in detail on the modelling techniques used.

The manuscript overall is well structured and well written. I only really therefore have three suggestions:

The manuscript could be shortened substantially, in particular the introduction. I would suggest cutting the text on lines 117 – 126 about ‘co-production’ and then lines 164 – 187 and instead replace with concise aims of the project which I interpret to be: i) identification of risk factors to include in models through stakeholder engagement; ii) development of predictive models; iii) validation of models. I do think the text/ framing around ‘co-production’ over complicates the paper and instead reference to ‘one-health’ approaches would suffice and simplify.

Response: We respectfully disagree since co-production provides a key new method for bridging the disconnect between zoonotic disease models, management and the One Health initiative. It provides a framework for integrating the needs and knowledge of cross-sectoral stakeholders and we would like to make more epidemiological modellers and practitioners aware of this approach through this paper. We notice the Reviewer 2# concurs that:

*“The inclusion of stakeholders is a useful model for conducting an investigation of this type, and the partnership appears to have been a fruitful collaboration among investigators from different areas of research.”*

In relation to the above, suggest changing the title to be explicit about what was done and what the study found. Perhaps something along the lines of ‘Spatial models developed through stakeholder engagement predict high risk areas of KFD in India’.

Response: We feel that a change to this title over-emphasises the predictive ability of the models, in light of our new analysis with full case data for the 2018-2019 season and neglects co-production as a novel One Health approach to developing predictive models

For figures showing predictive maps and human case locations perhaps have open circles or show cases separately on a 4th panel so that the map beneath can be seen clearly.

Response: See response to Reviewer #1 comments above.

Reviewer #2: Article is well-written overall. However certain portions in the results section needs to be shortened to avoid repetition with data presented in figures and tables. Longer sentences may be may be reframed into shorter sentences.

Response: We revised the manuscript to remove repetition and to break longer sentences into shorter ones.

Unnecessary use of brackets (such as 349, 382 ) may be avoided to maintain the flow of the sentence.

Response: We have removed unnecessary brackets throughout.
