# Supplementary material for: Predicting disease risk areas through co-production of spatial models: The example of Kyasanur Forest Disease in India’s forest landscapes
Source: PLoS Negl Trop Dis. 2020 Apr 7;14(4):e0008179. doi: 10.1371/journal.pntd.0008179 (PMC7164675; doi:10.1371/journal.pntd.0008179)
Supplement: Attachment — Submitted filename: Reviewers_comments_24022020.docx [file pntd.0008179.s011.docx]

Response to reviewers

We highly appreciate the time taken by the two reviewers and the editors to review the revised manuscript and their constructive comments. We are pleased that they felt that the manuscript had improved during the revision process. Here we itemise our response to each of the reviewer’s comments in blue under the original comments.

Reviewer #1: Check for consistency in unit placement after values e.g. line 249: 2km Line 223: 330 m

We have checked and now leave a space between the values and the units throughout.

Line 265: remove full stop

Full stop now removed

Table 1: all column contents start with capital letter except ‘proximity to health centre…’

This has been corrected.

Table 2: again, consistency with use of capitals, would also have each text in column two align with top of text relevant to each row in column three

We have checked consistency of capitals, capitalising column 2 variable names but not variable descriptions in column 3. The variable name and descriptions are now aligned as suggested.

Line 382: remove comma after full stop.

This has been corrected.

Line 443: Should be Liverpool School of Tropical Medicine (no Hygiene)

This has been corrected.

Table 3: again consistency with use of capitals

This has been corrected.

Line 557: insert 'be' in ‘is often predicted to present’

Reviewer #2: Minor edits suggested-

This has been corrected.

Line 381 - 'adhoc' to be changed to 'ad hoc', with addition of intervening space

This has been corrected.

Line 382 - space to be added after 'selected'

This has been corrected.

Line 382 - Consider removing comma immediately after full-stop.

This has been corrected.

Line 385 -386 (choice of punctuation marks) - a '-' and ','can be used instead of colon and semi-colon respectively.

This has been corrected.

Line 421 - remove space between 'to-' and 'expected' , thereby making 'predicted-to-expected' in continuity.

This has been corrected.

Lines 517-521 - the frequent use of semi-colon (;) in the sentence needs to be reconsidered. For example, there appears to be no need of semi-colon after 'when' in line 517. Other semi-colon may be replaced by comma.

We agree and have replaced semi-colons with commas in this instance.

Lines 688-692 - inclusion of '(1)' and '(2)' within the sentence are not necessary.

We have shorted the beginning of the sentence but kept the numbering for clarity.

Line 756-761 - The last sentence in discussion section appears to be an inordinately long sentence. Needs to be re-framed/ shortened.

We have split up and reframed this sentence.

Similarly 688-692 and 676-680 are examples where the sentence used appears to be quite long. It appears to me that average sentence length is longer in this article than what would be needed for easy comprehension by readers.

We have shortened both these sentences and made efforts to shorten or split up long sentences where-ever possible throughout the manuscript.

Likewise, authors are suggested to review the manuscript for minor corrections and proper presentation of text.

We have reviewed the manuscript for minor typos and text presentation (in the accepted changes version) and broken up or rephrased long sentences.

--------------------

Reviewer #1: I accept the authors points about 'co-production' in repsonse to my initial comments. I do not have any further comments and think that this draft is suitable for publication.

Reviewer #2: The suggested changes have been incorporated. Where not incorporated, proper justification has been provided. Manuscript is much improved as a result.

Authors have done well to have a comprehensive data analysis. Since the write-up is long, the possibility for minor errors and consequently, the scope for improvement is also present. However, a small additional effort in making the write-up crisp and free from minor errors will boost the quality of the article.

We have reviewed the manuscript for minor typos and text presentation (in the accepted changes version) and broken up or rephrased long sentences.

Please note that the figures and supplementary files have not been updated in this version since none of the reviewers’ comments were pertaining to the figures or these files.
